# Comparative Physiological and Transcriptome Analyses of Tolerant and Susceptible Cultivars Reveal the Molecular Mechanism of Cold Tolerance in *Anthurium andraeanum*

**DOI:** 10.3390/ijms25010250

**Published:** 2023-12-23

**Authors:** Na Dou, Li Li, Yifu Fang, Shoujin Fan, Chunxia Wu

**Affiliations:** 1 Shandong Provincial Key Laboratory of Plant Stress, College of Life Sciences, Shandong Normal University, Wenhua East Road 88, Jinan 250014, Chinafansj@sdnu.edu.cn (S.F.); 2 Institute of Ornamental Plants, Shandong Provincial Academy of Forestry, Wenhua East Road 42, Jinan 250010, China; fyf7741@163.com

**Keywords:** *Anthurium andraeanum*, cold stress, transcriptome, WGCNA, plant hormone signal, trehalose, ribosomal protein

## Abstract

*Anthurium andraeanum* is a tropical ornamental flower. The cost of *Anthurium* production is higher under low temperature (non-freezing) conditions; therefore, it is important to increase its cold tolerance. However, the molecular mechanisms underlying the response of *Anthurium* to cold stress remain elusive. In this study, comparative physiological and transcriptome sequencing analyses of two cultivars with contrasting cold tolerances were conducted to evaluate the cold stress response at the flowering stage. The activities of superoxide dismutase and peroxidase and the contents of proline, soluble sugar, and malondialdehyde increased under cold stress in the leaves of the cold tolerant cultivar Elegang (E) and cold susceptible cultivar Menghuang (MH), while the soluble protein content decreased in MH and increased in E. Using RNA sequencing, 24,695 differentially expressed genes (DEGs) were identified from comparisons between cultivars under the same conditions or between the treatment and control groups of a single cultivar, 9132 of which were common cold-responsive DEGs. Heat-shock proteins and pectinesterases were upregulated in E and downregulated in MH, indicating that these proteins are essential for *Anthurium* cold tolerance. Furthermore, four modules related to cold treatment were obtained by weighted gene co-expression network analysis. The expression of the top 20 hub genes in these modules was induced by cold stress in E or MH, suggesting they might be crucial contributors to cold tolerance. DEGs were significantly enriched in plant hormone signal transduction pathways, trehalose metabolism, and ribosomal proteins, suggesting these processes play important roles in *Anthurium*’s cold stress response. This study provides a basis for elucidating the mechanism of cold tolerance in *A. andraeanum* and potential targets for molecular breeding.

## 1. Introduction

Plants are sessile organisms, and during their life cycle, they encounter several abiotic stresses caused by harsh climatic changes. Cold stress is a detrimental abiotic stress affecting the growth and development, reducing the productivity and quality, and limiting the geological distribution of plants [1,2,3]. For example, low temperature stress can lead to plasma membrane peroxidation caused by reactive oxygen species (ROS), thus causing damage to internal cell structures [4,5]. To cope with cold stress, plants have developed complex strategies, including changes in a variety of molecular, physiological, and biochemical pathways [6]. During exposure to cold, the activities of antioxidant enzymes such as superoxide dismutase (SOD) and peroxidase (POD) and the contents of proline, soluble sugars, and proteins change; therefore, they are considered as physiological indicators of cold response in plants [7,8]. Genotypes with higher amounts of these compounds usually show cold resistance [9,10].

Sugars play an essential role in plant response to cold stress. Trehalose, a nonreducing disaccharide, has been detected in a wide range of organisms, including bacteria, fungi, invertebrates, and plants [11]. It not only serves as an energy source and a protectant but also functions as a signaling molecule in plants [12,13]. Trehalose is only present in trace amounts under optimal conditions, and its production increases in response to stresses [13,14]. In plants, trehalose is synthesized from UDP-glucose and glucose 6-phosphate in a reaction catalyzed by trehalose-phosphate synthase (TPS), and trehalose-phosphate phosphatase (TPP)and is degraded into glucose by trehalase [11]. Overexpression of TPS or TPP can improve the stress tolerance of transgenic plants [15,16]. Trehalose-6-phosphate (T6P), the precursor of trehalose biosynthesis in plants, is also a signaling molecule and interacts with SUCROSE-NON-FERMENTING1-RELATED KINASE1 (SnRK1) to balance sucrose levels in plants [17,18].

Numerous studies have demonstrated that plant hormones play important roles in regulating signal transduction in response to cold stress [2,19]. The plant hormone abscisic acid (ABA) is essential for inducing stomatal closure, seed dormancy, and abiotic stress response [20]. Cold stress can increase the endogenous ABA levels in plants, leading to increased cold resistance [21,22]. There are also ABA-independent signaling pathways involved in the response to low temperature stress. For example, cold stress affects the transport of auxin and inhibits the intracellular trafficking of auxin efflux carriers [23]; this in turn affects organogenesis and morphogenesis, which are regulated by the accumulation and polar distribution of auxin in plant tissues [24]. Other phytohormones, such as brassinosteroid (BR) [25], jasmonic acid (JA) [26], ethylene [27], and salicylic acid (SA) [28], also play a critical role in plant cold stress response.

Low temperature also induces a change in gene transcription and protein synthesis. For example, the expression pattern of ribosomal proteins changes during plant response to low temperature, thereby affecting mRNA translation and protein synthesis. The accumulation of three soybean ribosomal proteins (GmRPS13, GmRPS6, and GmRPL37) was found to be induced by low temperature [29]. Plastid ribosomal proteins, which are essential components of protein synthesis machinery, also have diverse roles in plant growth, development, and response to abiotic stress, especially temperature stress [30]. For example, chilling stress was found to cause ribosomal pausing during photosystem II component-D1 protein elongation in tomato [31]. Rpl33, a ribosomal protein that does not affect plant viability and growth under standard conditions, is required for sustaining plastid translation under cold stress [32]. Loss of function of the ribosomal subunit protein S5 (RPS5), a plastid 30S subunit, was found to affect photosynthesis proteins and cold stress response, possibly via a reduction in plastid translational capacity [33]. Plants with mutations in *TCD11*, which encodes RPS6 in chloroplasts, grow normally at 32 °C but die at 20 °C, indicating that TCD11 functions in chloroplast development at low temperature [34]. All these findings suggest that cold stress has the potential to impact virtually all plant processes by affecting protein translation.

*Anthurium andraeanum* (hereafter *Anthurium*) is a tropical ornamental plant known for its colorful spathe (i.e., leaf-like bracts enclosing the flowers) and spadix (inflorescence containing small flowers). *Anthurium* is commercially produced worldwide to be sold as cut flowers or as a potted plant. It does not grow well when the temperature decreases below 12 °C [35]. Therefore, *Anthurium* is often grown in greenhouses in north and northeast China, and supplemental heating is necessary in late autumn, winter, and early spring, which increases energy consumption and production costs. Therefore, it is of great interest to elucidate the molecular mechanism of *Anthurium*’s response to cold stress, to enable the development of cold-tolerant *Anthurium* varieties. 

Studies aimed at elucidating such mechanisms have identified genes potentially involved in the response of *Anthurium* to cold stress. De novo transcriptome analysis of 3-month-old *Anthurium* cv. Alabama seedlings subjected to 6 °C temperature revealed a number of cold-inducible transcription factors and pathways in *Anthurium* [36]. Thereafter, *AnAPX* [35], *AabHLH35* [37], and *microRNA158* [38] were found to function in the response to cold stress. In addition, exogenous application of chemical agents, γ-aminobutyric acid (GABA) [39], and putrescine [40] have been shown to alleviate chilling injury in *Anthurium*. Although these reports have provided insight into cold tolerance in *Anthurium*, the molecular mechanisms underlying the response to low temperature stress are not clear and need to be further investigated. 

In a preliminary study (unpublished data), the cultivars of *Anthurium* plants were collected from a producer in Shandong Province, China, and the cold responses of these plants were evaluated. Two cultivars, one cold-tolerant and the other cold-sensitive, were selected for further study. In this study, the morphological and physiological changes of these two cultivars when exposed to cold conditions were assessed. Then, the transcriptome profiles of the two cultivars under cold stress at the full flower phase were determined. The cultivar-specific genes and pathways and those common to both were revealed from analysis of the transcriptome data. This study provides insight into the mechanisms underlying *Anthurium*’s cold-stress response, thus facilitating the development of cold-tolerant *Anthurium* varieties.

## 2. Results

### 2.1. Cold Stress Triggers Morphological Differences between E and MH

**Figure 1:**
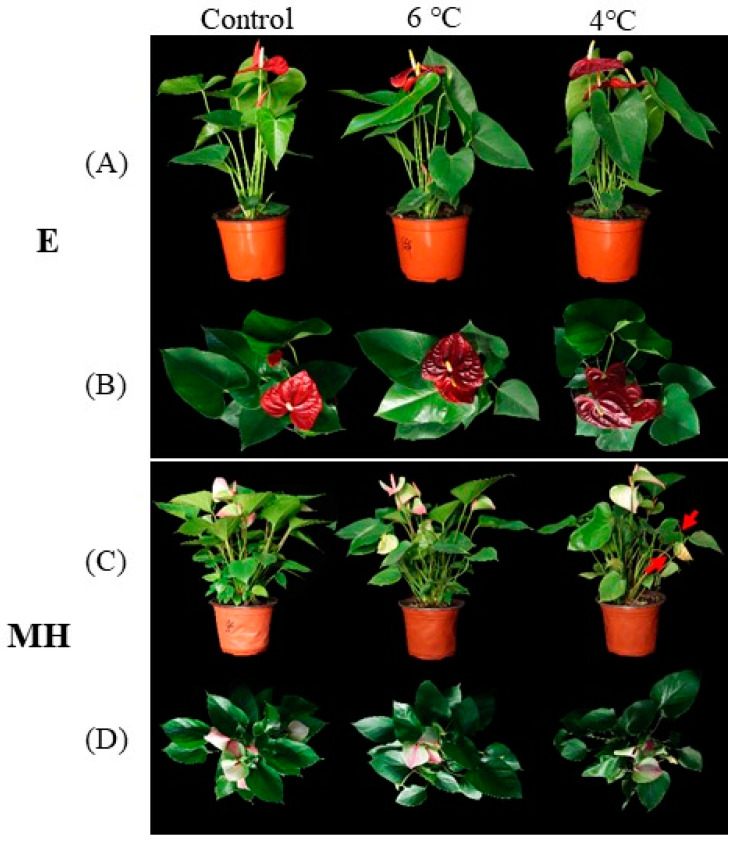
Phenotypic responses of E and MH to cold stress. Images of E and MH grown at 25/22 °C (control) then kept under the same conditions or transferred to 6 °C or 4 °C for 3 days. (**A**,**C**) Side views of the plants and (**B**,**D**) top views of the plants. The red arrow indicates the softened petiole of MH.

To compare cold responses among different *Anthurium* cultivars, we monitored the growth of 16 *Anthurium* cultivars at different temperatures as a preliminary study (unpublished data). Two *Anthurium* cultivars, Elegang (E, cold-tolerant) and Menghuan (MH, cold-susceptible), were selected to further examine the differences in the responses of *Anthurium* cultivars to cold stress. These cultivars are the most popular flowers in the Chinese market. When the *Anthurium* plants were subjected to 6 °C for 3 days, portions of the E spathes became deep red, but there was no visible change in the color of MH spathes (Figure 1B,D, 6 °C). When the plants were grown for 3 days at 4 °C, the E spathes were all deep red (Figure 1B, 4 °C), while the petioles of some young leaves of MH began to soften and the leaves began to droop (Figure 1C, red arrow). These phenotypic results show that MH is more susceptible to cold stress than E.

### 2.2. Cold Stress Affects the Physiological Responses of E and MH

**Figure 2:**
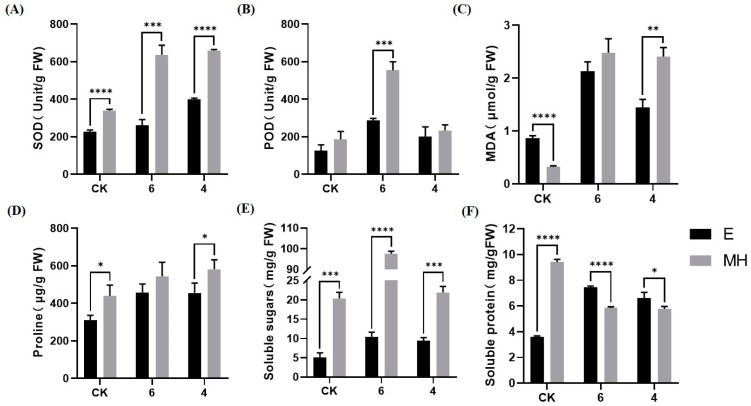
Differential physiological responses of E and MH to cold stress. (**A**) Superoxide dismutase (SOD) activity, (**B**) peroxidase (POD) activity, and the contents of (**C**) malondialdehyde (MDA), (**D**) proline, (**E**) soluble sugar, and (**F**) soluble protein. Error bars represent SD (*n* = 3). The statistical significance was determined by multiple *t*-tests. *p* < 0.05, *; *p* < 0.01, **; *p* < 0.001, ***; *p* < 0.0001, ****.

To further evaluate the cold tolerance of these two *Anthurium* cultivars, the physiological responses were measured in the leaves of control and cold-treated plants. The activities of SOD and POD and the contents of osmo-protectants (proline, soluble sugar, and soluble protein) at 25/22 °C (control conditions) were higher in MH than in E (Figure 2A,B,D,E,F). The change trends in SOD activity, POD activity, and the contents of proline and soluble sugars in response to cold treatment were similar in the two cultivars, but the levels of these parameters in E were lower than those in MH (Figure 2A,B,D,E). 

Cold stress induces ROS accumulation in plant cells, and antioxidative enzymes are the key ROS scavengers [41]. The activities of SOD and POD increased in both cultivars under cold stress (Figure 2A,B). However, the POD activity in MH was almost three-fold higher at 6 °C than at 25/22 °C, though the level at 4 °C was the same as that at the control temperature (Figure 2B). The content of MDA, which is an important marker of lipid peroxidation and membrane damage [42,43], increased about 16-fold in MH at 6 °C compared with the control and was higher than that of E after cold treatment (Figure 2C), indicating that E had a higher capacity to cope with oxidative stress than MH. Osmo-protectants including proline, soluble sugars, and soluble proteins accumulate in cells to protect against cell damage [44]. The proline content increased in response to cold stress, and in both cultivars it did not significantly differ between the 6 °C and 4 °C treatments (Figure 2D). The soluble sugar contents were higher at 6 °C compared with the control for both varieties, but those in MH were 4.8-fold higher while those in E were only 2.2-fold higher (Figure 2E). No significant difference was observed in soluble sugar content between the control and the treatment at 4 °C (Figure 2E). Interestingly, the soluble protein content of MH decreased under cold stress relative to the control, while that of E increased (Figure 2F). Consistent with the phenotypic results, the physiological data indicated that MH is sensitive to cold stress and E is cold-tolerant.

### 2.3. RNA Sequencing and De Novo Transcriptome Assembly

**Figure 3:**
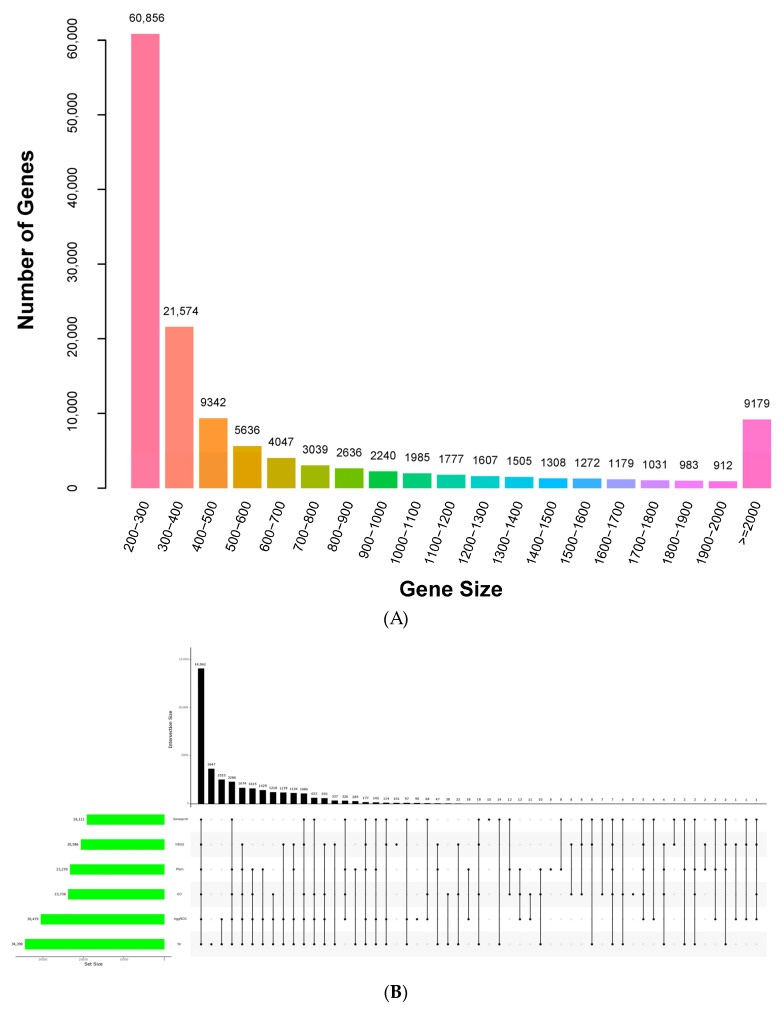

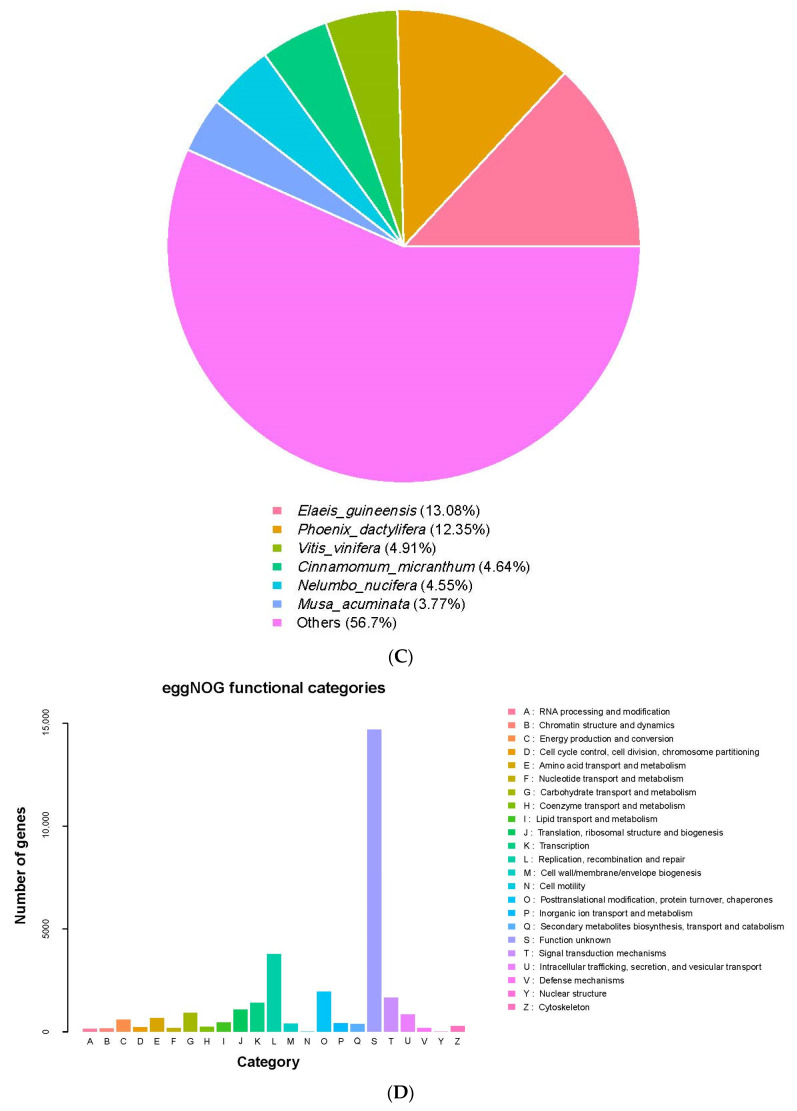
Overview of RNA sequencing results and annotation of unigenes. (**A**) Distribution of unigenes. (**B**) Overlap between the number of all unigenes according to six databases. (**C**) Distribution of unigene annotations based on the Nr database for the species distribution statistics. (**D**) eggNOG functional classification of all unigenes.

Bliss et al. (2012) estimated the genome size of *Anthurium* to be 4.6 Gb based on the bulk nuclear DNA contents for 77 accessions determined by flow cell cytometry [45]. To date, no genome sequence of *A. andraeanum* has been reported. Through de novo transcriptome analysis, genetic information for organisms can be obtained without genomic information [46], novel genes regulating pathways can be discovered, and the expression patterns can be elucidated. Eighteen cDNA libraries were constructed using the RNA extracted from the leaves of E (cold-tolerant) and MH (cold-sensitive) incubated at 25/22 °C (E_CK and MH_CK), 6 °C (E_6 and MH_6), and 4 °C (E_4 and MH_4) for 3 days. The libraries were sequenced on an Illumina Novaseq™ 6000. Sequencing generated a total of 129.44 Gb of raw reads for all 18 samples and an average of 7.19 Gb of reads for each sample (Table S1). After processing, 117.67 Gb of the clean reads were generated with an average Q30 of 95.09% (Table 1). The Trinity assembler generated 132,108 unigenes with a minimum length of 201 bp (Table 1, Figure 3A). The lengths of unigenes varied from 201 bp to 16,127 bp with an average length of 648 bp and an N50 length of 1207 bp, and the GC content was 44.84% (Table 1, Figure 3A). The clean reads were assembled into 290,721 transcripts with lengths ranging from 200 bp to 16,127 bp (Table 1).

### 2.4. Functional Annotation of Unigenes

To determine their possible functions, the 132,108 unigenes were annotated using six databases (NCBI Nr, SwissProt, Gene Ontology [GO], Kyoto Encyclopedia of Genes and Genomes [KEGG], Pfam, and eggNOG). A total of 34,390 unigene sequences had matches in the NCBI Nr database (26.03%), 30,479 in eggNOG (23.07%), 23,730 in GO (17.96%), 23,270 in Pfam (17.61%), 20,586 in KEGG (15.58%), and 19,112 in SwissProt (14.47%), with the most matches in the NCBI Nr database (Figure 3B). The unigenes annotated in the Nr database can reflect the species distribution statistics. The *Anthurium* unigene sequences showed the highest similarity to sequences from *Elaeis guineensis* (13.1%), followed by *Phoenix dactylifera* (12.35%), *Vitis vinifera* (4.91%), *Cinnamomum micranthum* (4.64%), *Nelumbo nucifera* (4.55%), and *Musa acuminata* (3.77%) (Figure 3C).

In the eggNOG database, the annotated unigenes were divided into 23 categories (Figure 3D). “Replication, recombination, and repair” (3833, 12.58%), “posttranslational modification, protein turnover, chaperones” (1951, 6.40%), and “signal transduction mechanisms” (1668, 5.47%) were the top three categories in terms of numbers of annotated genes. The categories with the fewest unigenes were “cell motility” (6, 0.020%) and “nuclear structure” (1, 0.003%).

To analyze the functions of the unigenes, the GO database annotations were classified in three main ontologies: biological process (BP, 19,884, 83.79%), molecular function (MF, 20,472, 86.27%), and cellular component (CC, 21,085, 88.85%) (Table S2). Unigenes were annotated to 4810 BP terms, and the term with the most annotations was “regulation of transcription, DNA-templated” (1365, 6.86%). For CC, “nucleus” (6677, 31.67%) and “cytoplasm” (3945, 18.71%) were the most abundant among the 970 terms, while for MF, “protein binding” (2665, 13.02%) and “ATP binding” (1731, 8.46%) were the two most frequent terms (Figure S1). A total of 20,586 unigenes was clustered into 20 KEGG pathway categories (Figure S2). The most significant KEGG pathway was “carbohydrate metabolism” (1623, 7.88%), followed by “translation” (1500, 7.29%) and “folding, sorting, and degradation” (1346, 6.54%) (Figure S2).

### 2.5. Differential Expression Analysis of Anthurium Genes

**Figure 4:**
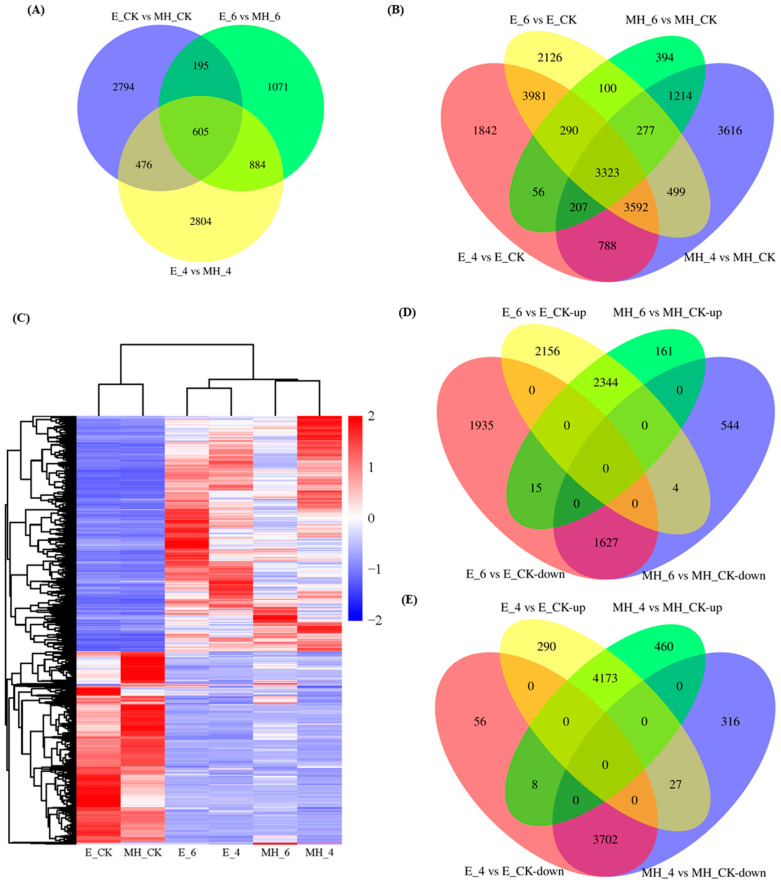
DEGs of two cultivars in response to cold stress. (**A**) Venn diagram showing DEGs’ profile between E and MH under different cold treatments. (**B**) Venn diagram showing the overlap in DEGs in both cultivars between cold and control conditions. (**C**–**E**) The 9132 common DEGs differentially expressed in both cultivars during cold stress. (**C**) Heatmap showing the results of clustering analysis of the 9132 common CSR DEGs. (**D**) Venn diagram showing the overlap in DEGs expressed after 6 °C cold stress in both cultivars. (**E**) Venn diagram showing the overlap in DEGs expressed under 4 °C cold stress in both cultivars.

To dissect the different cold-stress responses of the two *Anthurium* cultivars at the transcript level, DEGs were identified between cultivars under control (CK) and cold-stress conditions (4 or 6 °C) using the criteria |log_2_FC| > 2 and FDR < 0.001. A total of 8829 DEGs were identified between E and MH: 4070 DEGs were detected from the comparison of E_CK and MH_CK (1682 up and 2388 down), 2755 DEGs from E_6 and MH_6 (1474 up and 1281 down), and 4769 DEGs from E_4 and MH_4 (2600 up and 2169 down) (Figure 4A and Figure S3A, Table S3). These results indicated that many genes are differentially expressed between the two cultivars under control and cold-stress conditions.

Gene expression differences in both cultivars between control vs. cold (6 °C and 4 °C treatments) were calculated. A total of 22,305 DEGs were obtained from comparisons 6 °C versus CK and 4 °C versus CK for both cultivars: 14,188 (8616 up and 5572 down) and 5861 (3203 up and 2658 down) DEGs were identified between 6 °C and CK for E and MH, respectively, and 14,079 (8166 up and 5913 down) and 13,516 (6880 up and 6636 down) DEGs were identified between 4 °C and CK for E and MH, respectively (Figure 4B and Figure S3B, Table S4). There were more upregulated genes than downregulated genes in all comparisons (Figure S3B). At 6 °C, there were more DEGs in the cold-tolerant cultivar E than in the cold-sensitive cultivar MH (Figure S3B), which indicated that more genes show changes in expression to response to cold in E than in MH in the early stage of cold stress.

The 22,305 DEGs were assigned to two groups based on the overlap between comparisons: (1) genotype-specific cold-stress-responsive (CSR) DEGs differentially expressed between the control and cold treatment in only one cultivar and (2) common CSR DEGs differentially expressed in both cultivars. There were 2126 and 1842 DEGs in the E_6 vs. E_CK and E_4 vs. E_CK comparisons, respectively, and there were 3981 DEGs common to E_6 vs. E_CK and E_4 vs. E_CK. Thus, 7949 DEGs were specific to the cold-tolerant cultivar E (Figure 4B). Similarly, there were 394 and 3616 DEGs in the MH_6 vs. MH_CK and MH_4 vs. MH_CK comparisons, respectively, and there were 1214 DEGs common to MH_6 vs. MH_CK and MH_4 vs. MH_CK. Thus, 5224 DEGs were specific to the cold-sensitive cultivar MH. There were 9132 (100, 290, 56, 277, 3323, 207, 499, 3592, 788) common CSR DEGs identified in the 6 °C vs. CK and 4 °C vs. CK comparisons for both cultivars (Figure 4B, Table S5). The identification of common CSR DEGs indicat that there are conserved cold-stress responses in the two cultivars. However, further analysis of these genes showed that the expression patterns of these genes were inconsistent between the two cultivars under 6 °C and 4 °C stress (Figure 4C). These common DEGs were further analyzed to investigate the differences in their expression patterns between the two cultivars.

Among these 9132 common CSR DEGs, when comparing 6 °C with CK, there were 8081 DEGs (4504 upregulated and 3577 downregulated genes) in E and 4695 DEGs (2520 upregulated and 2175 downregulated genes) in MH (Figure 4D); whereas, at 4 °C, there were 8256 DEGs (4490 upregulated and 3766 downregulated genes) in E and 8686 DEGs (4641 upregulated and 4045 downregulated genes) in MH (Figure 4E). Among the common CSR DEGs, more genes were upregulated under cold stress than downregulated.

All the CSR DEGs common to both varieties and both cold treatments were compared. When the *Anthurium* plants were treated at 6 °C for 3 days, 2156 DEGs were upregulated and 1935 DEGs were downregulated exclusively in E, while 161 and 544 DEGs were specifically upregulated and downregulated in MH, respectively (Figure 4D). However, 4 DEGs were upregulated in E and downregulated in MH, while 15 DEGs were downregulated in E and upregulated in MH. A total of 2344 DEGs were upregulated while 1627 DEGs were downregulated in both E and MH. Under the 4 °C treatment, 290 DEGs were upregulated and 56 DEGs were downregulated only in E, while 460 DEGs were upregulated and 316 DEGs were downregulated only in MH (Figure 4E). Twenty-seven DEGs were upregulated in E and downregulated in MH, and eight DEGs showed the opposite expression patterns. Furthermore, 4173 and 3702 DEGs were upregulated and downregulated, respectively, in both cultivars. Under 6 °C stress, there were more DEGs specific to E than to MH, while at 4 °C there were fewer E-specific DEGs and significantly more up- and down-regulated DEGs in both cultivars compared with the 6 °C treatment. These results indicate that the gene expression changes enable the plant to cope with cold stress occurred at an earlier stage in E than in MH.

### 2.6. Functional Analysis of Common CSR DEGs in Anthurium

**Figure 5:**
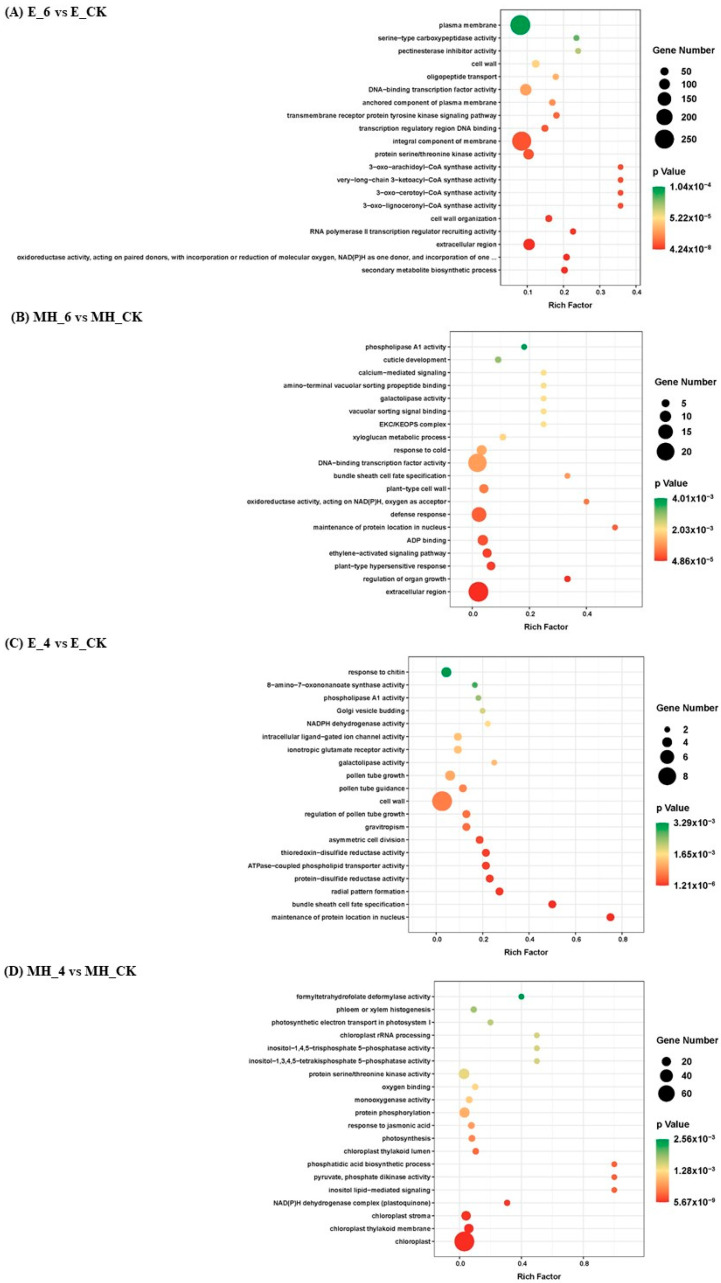
Gene Ontology enrichment analysis of different groups of the 9132 common CSR DEGs.

To characterize the functions of the DEGs, we performed GO and KEGG enrichment analysis using OmicStudio (https://www.omicstudio.cn/tool, accessed on 14 October 2023) for the DEG groups described above (Figure 5, Figures S4 and S5). Under 6 °C stress, DEGs (2156 up and 1935 down) specific to the cold-tolerant cultivar E were enriched in the GO terms “secondary metabolite biosynthesis”; “oxidoreductase activity, acting on paired donors, with incorporation or reduction of molecular oxygen, NAD(P)H as one donor, and incorporation of one atom of oxygen”; and “extracellular region”. Interestingly, some CoA synthases for long-chain fatty acid stood out significantly. KEGG analysis revealed enrichment in “pentose and glucuronate interconversions”, “fatty acid elongation”, and “phenylalanine metabolism”. For the cold-sensitive cultivar MH, the specific DEGs (161 up and 544 down) were enriched in the GO terms “extracellular region”, “regulation of organ growth”, and “plant-type hypersensitive response” as well as the KEGG pathways “plant hormone signal transduction”, “alpha-linolenic acid metabolism”, and “MAPK signaling pathway—plant”. This result indicated that changes in energy metabolism and membrane lipids occur in the cold-tolerant cultivar E, whereas signal transduction is affected in the cold-sensitive cultivar MH. After 4 °C stress, E-specific DEGs (290 up and 56 down) were enriched in “maintenance of protein location in nucleus”, “bundle sheath cell fate specification”, and “radial pattern formation” as well as the KEGG pathways “alpha-linolenic acid metabolism”, “biotin metabolism”, and “galactose metabolism”. The MH-specific DEGs (460 up and 316 down) were enriched in the GO terms “chloroplast”, “chloroplast thylakoid membrane”, “chloroplast stroma”, “NAD(P)H dehydrogenase complex (plastoquinone)” and “chloroplast thylakoid lumen”, which are the cellular components associated with photosynthesis. The enriched KEGG pathways were “carbon fixation in photosynthetic organisms”, “carotenoid biosynthesis”, and “nitrogen metabolism” (Figure 5). The enrichment of these categories suggests that photosynthesis and the carbon and nitrogen balance may be affected in MH. These results reflect differences in the responses of the two *Anthurium* varieties to cold stress.

DEGs in both varieties may reflect common mechanisms in the response of *Anthurium* to cold stress (Figure S5). Under 6 °C stress, the 2344 DEGs upregulated in both cultivars were enriched in trehalose-related terms (GO:0004805, GO:0003825, GO:0070413, and GO:0005992), “response to chitin”, “DNA-binding transcription factor activity”, and transcription-related terms. For the downregulated DEGs common to both cultivars, photosynthesis-related biological process and cellular component terms were enriched. Under 4 °C stress, the common upregulated DEGs in both cultivars were enriched in “response to chitin”, “sequence-specific DNA binding”, “regulation of defense response”, “protein phosphorylation”, and trehalose-related terms. For downregulated DEGs, the enriched terms were “cell wall organization”, “extracellular region”, and photosynthesis-related biological process and cellular component terms. The E-specific DEGs at 6 °C were also enriched in CoA synthase for long-chain fatty acid. These results suggest that trehalose metabolism is important in the response of *Anthurium* to cold stress and that photosynthesis-related processes are affected under cold stress.

Among the common CSR DEGs, four DEGs were upregulated in E and downregulated in MH under 6 °C stress (Figure 4D). One was a heat-shock protein, which is involved in protein processing in the endoplasmic reticulum (Table 2). These four DEGs may be critical in the response of E to cold stress. Fifteen DEGs were upregulated in MH and downregulated in E at 6 °C (Table 2). Three encoded a glutamate receptor (GLR), three encoded subtilisin-like proteases (SBTs), two encoded cysteine-rich receptor-like protein kinases (CRK), one encoded a BURP-domain containing protein, one encoded a zinc-finger protein, and one encoded transmembrane protein. After 4 °C treatment, there were 27 DEGs upregulated in E and downregulated in MH, encoding one heat-shock protein (HSP), one heat-stress transcription factor (HSF), three cell-wall-associated enzymes (pectinesterase or pectinesterase-like proteins), one putative nucleotidyltransferase, one fatty acid amide hydrolase-like (FAAH), one MYB transcription factor, and one chloroplastic early light-induced protein (ELIP1) (Table 3). The eight DEGs downregulated in E and upregulated in MH encoded GPAT6, a probable disease resistance protein, CRK, and CYC P450 (Table 3). These common CSR DEGs with the opposite expression patterns may play vital roles in *Anthurium* cold tolerance. Their functions need to be further studied to determine if they can be used as target genes to improve *Anthurium* cold tolerance. 

### 2.7. Co-Expression Network Analysis of DEGs

**Figure 6:**
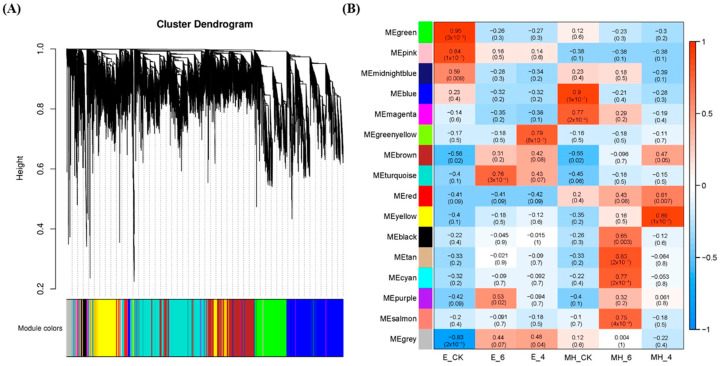
Identification of co-expression gene modules using DEGs. (**A**) The hierarchical cluster tree shows 16 co-expressed modules identified by WGCNA. Different modules are marked with different colors. (**B**) Module-cold treatment correlation. Each row represents a module. Each column corresponds to a specific sample. The color of each cell at the row–column intersection indicates the correlation coefficient between the module and sample. Red indicates a high degree of correlation between a specific module and the sample.

Weighted gene co-expression network analysis (WGCNA) is a systems biology method for describing the correlations among genes across samples [47]. WGCNA is used to not only construct gene networks but also detect gene modules and identify the central players (i.e., hub genes) within modules. To comprehensively decipher the mechanism of cold response in *Anthurium*, all DEGs (24,695) were used to build a gene co-expression network. Via sample clustering, 18 samples were included in the analysis. Based on pairwise correlations of gene expression, 16 modules were identified (marked with different colors in Figure 6A). A correlation heatmap of module-cold relationships was constructed to clearly visualize the correlation of modules and cold treatment (Figure 6B). The turquoise module was associated with the E_6 (*R* = 0.76, *p* = 3 × 10^−4^), and the greenyellow module was associated with E_4 (*R* = 0.79, *p* = 8 × 10^−5^). Importantly, these two modules were negatively associated with the MH samples. The tan and yellow modules were tightly associated with MH_6 (*R* = 0.83, *p* = 2 × 10^−5^) and MH_4 (*R* = 0.89, *p* = 1 × 10^−6^), respectively, and both modules were negatively correlated with the E samples.

In the turquoise module, the top enriched GO terms were “nucleus”, “plasma membrane”, and “cytoplasm” and the top KEGG pathways were “plant hormone signal transduction”, “plant-pathogen interaction”, and “MAPK-signaling pathway-plant”. The top enriched GO terms for the greenyellow module were “extracellular exosome”, “membrane”, and “structural constituent of ribosome”. The top three enriched KEGG pathways were “ribosome”, “oxidative phosphorylation”, and “glycolysis/gluconeogenesis”. These results indicat that signal transduction, plant immunity genes, protein synthesis, and degradation are important for resistance to cold stress in E. In the tan module, there were three top GO terms with the same DEGs and Q-values: “quercetin 3-O-glucosyltransferase activity”, “quercetin 7-O-glucosyltransferase activity”, and “flavonoid glucuronidation”. The top enriched KEGG pathways were “carotenoid biosynthesis”, “pentose and glucuronate interconversions”, and “MAPK signaling pathway—plant”. The top three GO terms for the yellow module were “nucleus”, “cytoplasm”, and “mitochondrion”, while the top KEGG pathways were “sphingolipid metabolism”, “starch and sucrose metabolism”, and “amino sugar and nucleotide sugar metabolism” (Figure S6, Table S6). These findings indicate that carotenoid biosynthesis and sugar metabolism may be involved in the response of MH to cold stress.

**Figure 7:**
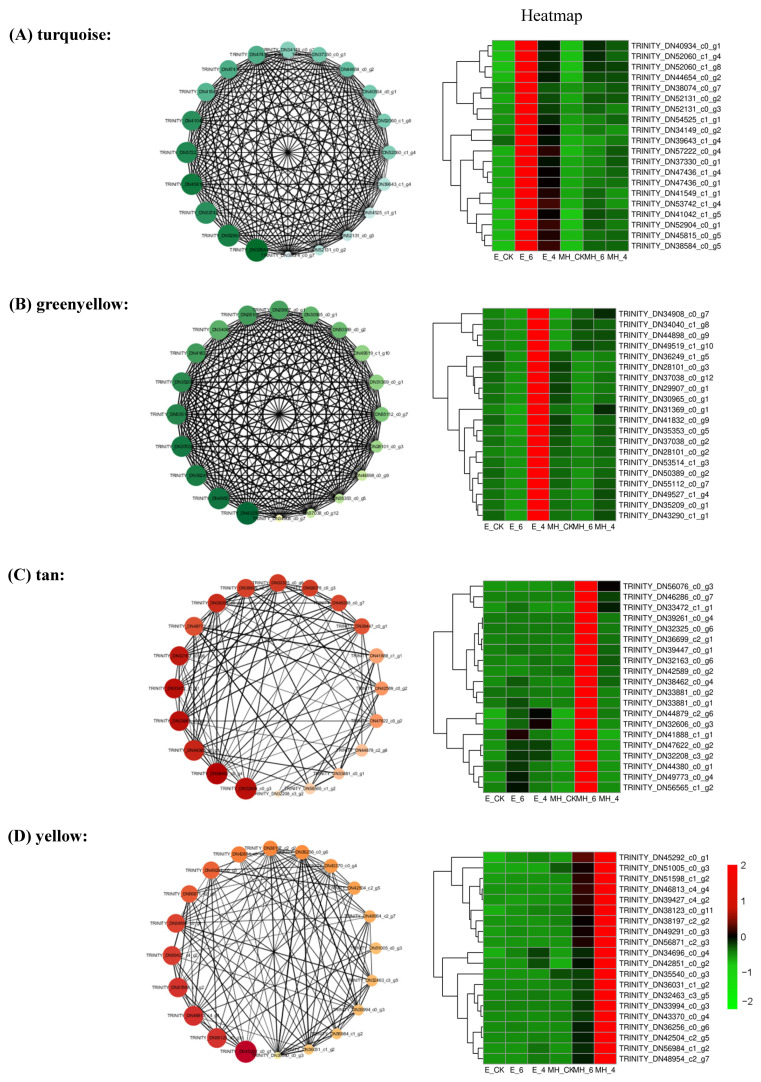
Identification of hub genes of the four key modules responsive to cold stress in *Anthurium*. The co-expression network of the top 20 hub genes of the key modules was visualized by Cytoscape. The heatmaps show the expression of the top genes of the four modules. (**A**) The turquoise module. (**B**) The greenyellow module. (**C**) The tan module. (**D**) The yellow module. The hub genes with the highest connectivity to other genes are shown in the nodes. The node colors corresponds to connectivity values, and their sizes represent the module membership value calculated by WGCNA. The darker and larger the node, the greater the hubness of the gene. The width of the lines between nodes corresponds to weight as calculated by WGCNA. The wider the line, the higher the weight value between the nodes. The values in the heatmap represent the z-score of the transcript levels in different samples. Red indicates a high expression level, while green is low.

The top 20 hub genes were identified by the cytohubba plug-in using the MCC algorithm in Cytoscape software. The edges with a weight value no less than 0.2 were used to calculate the top hub gene. In the turquoise module, *TIFY 10*, *MAN3*, *At1g32860* (glucan endo-1,3-beta-glucosidase 14-like), *AOS1* (allene oxide synthase 1, chloroplastic-like), *TL1* (thaumatin-like protein 1), and *FER* (receptor-like protein kinase ANXUR1) were included in the top 20 hub genes (Table S7). All these genes, except *FER*, have been found to be involved in abiotic and biotic stress responses in plants [48,49,50,51,52]. For the green-yellow module, the top 20 hub genes encoded nine ribosome proteins, three NADH dehydrogenase subunits, three elongation factors, two cytochrome oxidase subunits, an ATP synthase subunit, and fructose-bisphosphate aldolase (Table S7). This result indicates that protein and ATP synthesis are related to the response to cold. For the tan and yellow modules, most of the top 20 hub genes were not annotated in the NCBI database (Table S7). Three *Asp1* genes (encoding aspartic proteinases) were hub genes in the tan module (Table S7). The co-expression networks of these hub genes were visualized using Cytoscape (Figure 7). The top genes in the turquoise and greenyellow modules were remarkably upregulated in the E_6 or E_4 samples, while those in the tan and yellow modules had significantly high levels of expression in MH_6 and MH_4, respectively (Figure 7).

### 2.8. Plant Hormone Signal Transduction Is Involved in the Cold Stress Response in Anthurium

**Figure 8:**
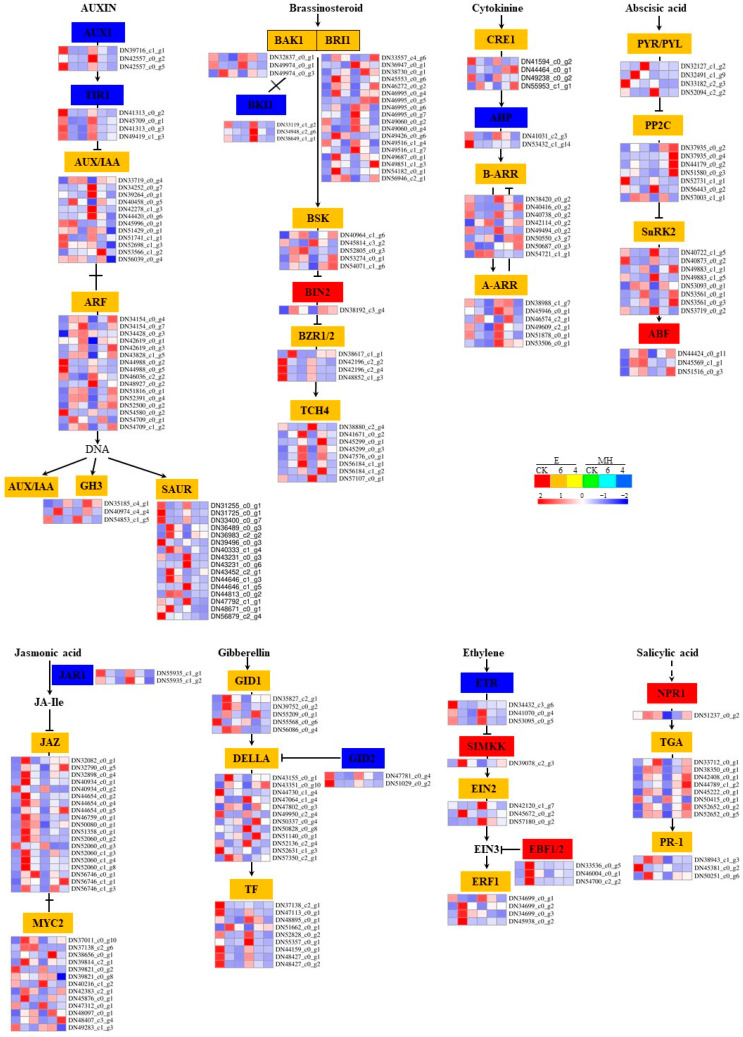
The expression patterns of DEGs in the “plant hormones signal transduction” pathway. Heatmap shows the expression of DEGs associated with different hormones in E and MH. In the pathways, blue indicates the downregulated genes, orange indicates down- and upregulated DEGs, and red indicates the upregulated genes. Mean TPM values of the three replicates were used to represent gene expression value in the heatmap.

Plant hormones play important roles in plant growth and plant response to stress [53]. In this study, there were 226 DEGs between treatment and control annotated to plant hormone signal transduction (Table S8), indicating that cold stress activates various plant hormone pathways (e.g., auxin, cytokinin, ethylene, ABA, JA, SA, BR, and GA) in *Anthurium* leaves (Figure 8, Table S8). The expression profiles of these 226 DEGs are shown in Figure 8. The auxin pathway genes encoding AUX1 and TIR1 were downregulated. Only 4 of the 12 AUX/IAA DEGs were upregulated. DEGs related to SAUR were all downregulated in MH, and the DEGs that were upregulated in response to cold in E showed no difference in MH. In both cultivars, negative regulators of the GA pathway were downregulated, and the downstream target genes were upregulated. All ethylene pathway DEGs, except ETR, were upregulated in E but downregulated in MH. There were more upregulated BR and JA pathway DEGs in E than in MH, and the number of DEGs encoding MYC2 and JAZ were especially high. The distinct expression patterns of these DEGs in E and MH reveal a complicated mechanism involving phytohormones under cold stress. Among the eight plant-hormone-related DEGs, most were related to auxin (54 DEGs), BR (42 DEGs), and JA (34 DEGs). Moreover, most of these DEGs were upregulated after cold stress. This suggests that low temperature induces the upregulation of genes related to phytohormones, to protect against cold injury and confer tolerance to cold stress in *Anthurium*.

### 2.9. Trehalose-Related Genes Are Induced in Anthurium in Response to Cold Stress

**Figure 9:**
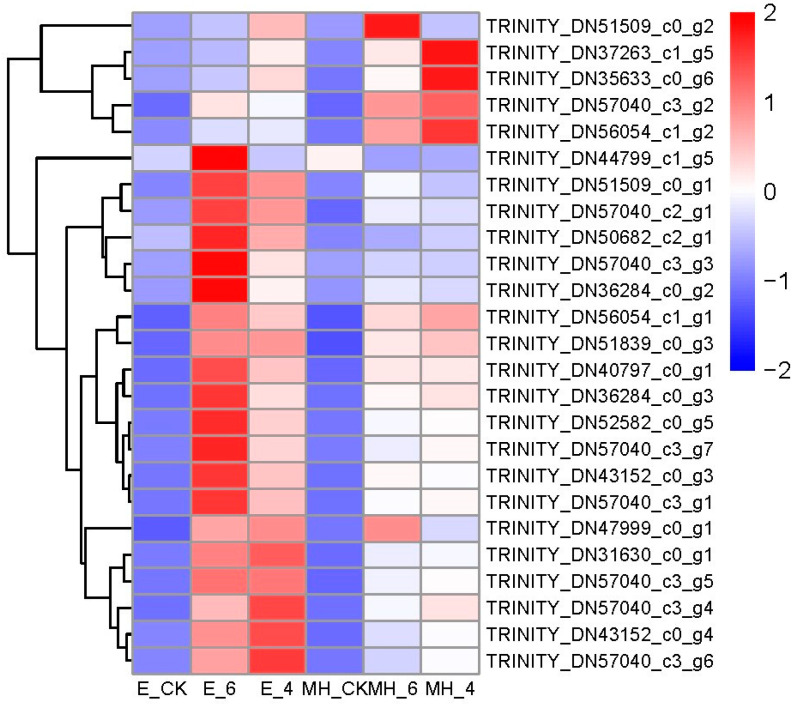
The expression patterns of 25 DEGs involved in trehalose metabolism. Mean TPM values of the three replicates were used to represent the gene expression value in the heatmap. The values in the heatmap represent the z-score of the transcript levels in different samples. Red indicates a high expression level, while blue is low.

Trehalose is a nonreducing disaccharide found in diverse organisms and serves as an energy source as well as an osmolyte and/or protein/membrane protectant [11]. Trehalose and its precursor, T6P, are involved in plant responses to multiple abiotic stresses [11]. TPS and TPP are the key enzymes in trehalose synthesis, while trehalase is the enzyme responsible for the degradation of trehalose. In this study, trehalose-related GO terms were significantly enriched in upregulated common CSR DEGs (Figure S5). We found 25 trehalose-related DEGs (22 TPS, 2TPP, and 1 trehalase) that were upregulated under cold stress (Figure 9, Table S9). Most of them were upregulated in E under cold stress but hardly or lowly expressed in the control and MH samples (Figure 9). 

### 2.10. Ribosomal Proteins Are Involved in Anthurium’s Response to Cold Stress

**Figure 10:**
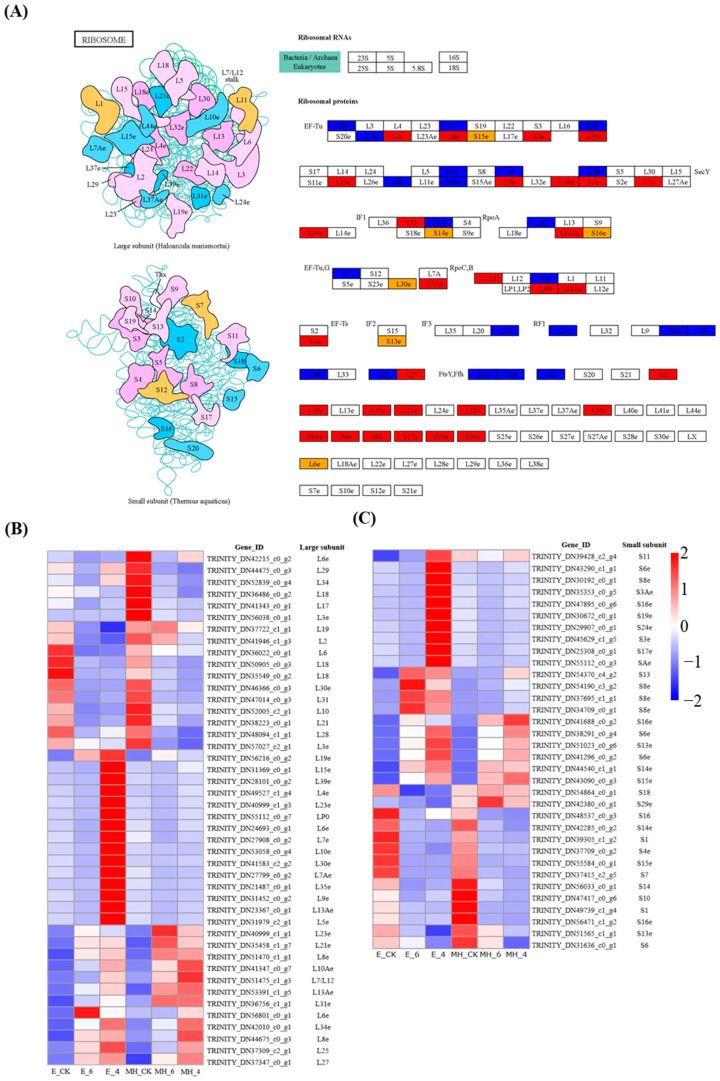
The expression patterns of DEGs encoding ribosome proteins (map03010). (**A**) The ribosomal DEGs. Blue indicates down-regulated genes, orange indicates down- and upregulated DEGs, and red indicates upregulated genes. (**B**) Heatmap of DEGs encoding large-subunit ribosomal proteins. (**C**) Heatmap of DEGs encoding small-subunit ribosomal proteins. In the heatmap, mean TPM values of the three samples were used to represent the gene expression value.

Ribosomes are the primary sites of translation and protein synthesis, and their main components are protein and rRNA. In the greenyellow module, ribosome was the top enriched KEGG pathway. Seventy-eight DEGs encoding ribosomal proteins were found to be differentially expressed in response to cold (Figure 10A, Table S10). These DEGs showed distinct expression patterns in the two cultivars under cold treatment (Figure 10B,C). There were more upregulated DEGs than downregulated DEGs, and there were more upregulated DEGs in E than in MH and more downregulated DEGs in MH than in E. Moreover, 25 upregulated DEGs were only found in the E_4 vs. E_CK comparison. These results suggest that mRNA translation and protein synthesis are more active in E (cold-resistant) than in the cold-sensitive variety MH, which is consistent with the differences in physiological indexes between these varieties.

### 2.11. Validation of DEGs by qRT-PCR

To validate the RNA-seq results, 16 common DEGs were randomly selected for qRT-PCR analysis. The qRT‒PCR data were generally consistent with the RNA-seq data, which confirmed the authenticity of the DEGs in this study (Figure S7).

## 3. Discussion

Low-temperature stress is detrimental and affects the growth and development, productivity, and distribution of plants. *Anthurium* is a tropical ornamental plant that is sold as cut flowers or potted plants. It is popular for its brightly colored spathe and spadix, so research has mainly focused on the mechanism giving rise to *Anthurium*’s spathe color and the environmental factors that affect color. There have been few studies on *Anthurium*’s response to cold stress, and the mechanism remains unclear. Tian et al. performed a time-series (1 h, 5 h, 24 h) transcriptome analysis of 3-month-old *Anthurium* cv. Alabama under 6 °C conditions [36]. Some transcription factors and pathways that may be involved in response to cold stress were identified. *AnAPX* gene cloned from *Anthurium* cv. Alabama that was upregulated under 6 °C stress was previously shown to enhance cold tolerance by preventing cell membrane damage under chilling stress [35]. *AabHLH35* was identified from transcriptome analysis of a *dark-green* leaf color mutant of *Anthurium* and was shown to improve the cold tolerance of transgenic *Arabidopsis* [37]. MicroRNAs have also been shown to be involved in regulating plant responses to cold stress. *Aa-miR158* was found to be expressed at higher levels in *Anthurium* under cold stress and to improve cold tolerance when overexpressed in *Arabidopsis* [38]. In this study, two *Anthurium* cultivars, E (cold-tolerant) and MH (cold-susceptible), were used as experimental materials. Transcriptome sequences were used to characterize the molecular mechanism of cold responses in *Anthurium*. The DEGs common to the different cultivars were analyzed, and the key modules and hub genes were identified through WGCNA. 

### 3.1. Morphological and Physiological Changes Differ between E and MH under Cold Stress

Low temperatures influence plant growth and development by causing various morphophysiological and biochemical changes [54,55]. In this study, the spathe color (red) of E was deeper after cold stress. For MH, the petiole of young leaves softened, and the leaf drooped (Figure 1). Therefore, E is resistant and MH is sensitive to cold stress.

Low-temperature stress causes metabolic imbalance and inhibits various cellular processes. Under cold stress, the photostatic imbalance induces cell lipid peroxidation and damage to the membrane structure, thereby influencing cell membrane fluidity and producing MDA, which is often used as a marker of lipid peroxidation and membrane damage [42,43]. The leaf MDA content in MH was higher than that of the control and of E after cold treatment (Figure 2C). These results indicate that the cell membrane in MH is damaged more severely than that in E under cold stress.

Antioxidant enzymes such as SOD and POD and osmolytes such as proline, soluble sugar, and soluble protein, usually play a key role in balancing ROS and the osmotic gradient in the cell [41,56]. Here, the activities of SOD and POD and the contents of proline, soluble sugar, and soluble protein were all higher in MH than in E under normal conditions. When plants grew under 6 °C stress, the activities of SOD and POD and the contents of proline and soluble sugars increased in both varieties compared with the control (Figure 2A,B,D,E), but the fold increases of soluble sugars were higher in MH (4.8-fold) than in E (2.2-fold) at 6 °C (Figure 2E). After the plants experienced 4 °C stress, the POD activity and the content of soluble sugars all decreased to control levels in E and MH (Figure 2B,E). These results indicate that SOD and POD play an important role in *Anthurium*’s response to cold stress and that POD activity could be inhibited under a lower temperature; they also indicate that proline and soluble sugars are involved in balancing the osmotic gradient between the cell’s surroundings and the cytoplasm under 6 °C stress. The soluble protein content of E increased while that of MH decreased under cold stress (Figure 2F). This indicats that soluble protein contributs to E’s cold resistance. MH was sensitive to cold even though the activities of antioxidant enzymes and the contents of osmolytes were higher than those in E under normal growth conditions. This suggests that it is the quick change in the biochemical indexes and not the starting levels of these indexes that is important in plant cold-resistance. 

### 3.2. Plant Hormone Signal Transduction Pathways Are Important for Cold Response in Anthurium 

Plant hormones function as vital regulators in the responses of plants to low temperatures [57,58,59]. Indole-3-acetic acid (a form of auxin), ABA, GA3 (a form of GA), JA, BR, and zeatin (a cytokinin) have been shown to play important roles in enabling plants to cope with stress, and an increase of plant hormones has been found to enhance resistance to cold stress in crop species such as rice [60], tomato [61], cucumber [62], and pear [63]. Common CSR DEGs and DEGs in the turquoise module were found to be enriched in genes involved in the KEGG plant hormone signal transduction pathway, and 226 DEGs were annotated to this pathway. In E, the upstream genes in the auxin and GA metabolic pathways were downregulated while the downstream genes were upregulated. More upregulated DEGs in the BR and JA metabolic pathways, especially DEGs related to JAZ, were found in E than in MH. This indicates that plant hormones and related genes play essential functions in the resistance of *Anthurium* to cold. The DEGs in this pathway may be good targets for further research.

### 3.3. Trehalose Metabolism in Anthurium Cold-Response

Trehalose is a disaccharide and serves as an energy source, protectant, and signal molecule in plants [11,12]. Trehalose accumulates and trehalose-related genes are induced during plant responses to abiotic and biotic stresses [13,14,64]. Recently, more and more studies have demonstrated that T6P, the intermediate of trehalose biosynthesis, functions as a signal and regulator in balancing sucrose levels in plants [17]. In this study, the upregulated common DEGs in both cultivars under cold stress (both 6 °C and 4 °C) were enriched in trehalose-related terms (GO:0004805, GO:0003825, GO:0070413, and GO:0005992). Analysis of the transcriptome data revealed that 25 DEGs are related to trehalose metabolism and upregulated after cold stress. These results indicat that an increase in trehalose metabolism may be a common plant response to cold stress and are consistent with the results of Mollavali et al. [64]. The DEGs encoding trehalose-related proteins could potentially be targeted to increase plant cold-stress tolerance.

### 3.4. Ribosomal Proteins Play Important Roles in Cold Tolerance in Anthurium 

The ribosome is the primary site for protein synthesis, and ribosomal proteins play vital roles in translation, ribosomal structure, and ribosome biogenesis. Ribosomal proteins also play roles in plant salt-stress responses [65,66], and several studies have implicated ribosome proteins involved in cold-stress response [29,31,32,33,34]. In this study, the soluble protein content in MH decreased under cold stress while that in E increased, and the greenyellow module identified by WGCNA contained 28 DEGs encoding ribosomal proteins. Seventy-eight genes encoding ribosomal proteins were identified as being differentially expressed in response to cold stress in *Anthurium* (Figure 10A, Table S10). More upregulated DEGs encoding ribosomal proteins were found in E than in MH. The expression patterns of ribosomal protein DEGs were consistent with the changes in soluble protein contents in the two *Anthurium* varieties. This suggests that the cold-resistant variety enhances protein synthesis in order to cope with the cold conditions, whereas the susceptible one cannot maintain protein synthesis, leading to a decrease in protein content.

### 3.5. HSP, Pectinesterase, and Plant Defense Proteins Are Commonly Involved in Anthurium Responses to Cold Stress 

Common DEGs between cold treatment and the control in different comparisons may reflect common cold-response mechanisms in *Anthurium*. HSPs and HSFs regulate protein folding and have multiple functions in abiotic stress [67,68]. For example, small HSPs (sHSPs) exhibit chaperone activity [69], and heterologous expression of a chestnut sHSP enhanced *Escherichia coli* viability under cold stress [70]. *GbHSP16.8* and *GbHSP17* were found to be induced by cold stress in *Ginkgo biloba* [71]. Transgenic *Arabidopsis thaliana* constitutively expressing *PfHSP17.2*, as isolated from *Primula forrestii* leaves, displayed higher resistance to cold compared with wild-type plants [72]. Song et al. (2016) reported that the expression of HsfA3 was induced by oxidative damage in *Arabidopsis* [73]. HsfAs of bermudagrass were upregulated at 5 °C [68]. In this study, two sHSPs and one HsfA were among the common DEGs upregulated in E and downregulated in MH (Table 2 and Table 3), indicating that sHSPs and HsfA may be involved in establishing cold tolerance in *Anthurium*. 

The cell wall is a dynamic polysaccharide network that offers the plant stability and protection under cold stress. Pectinesterases, which are ubiquitous cell-wall-associated enzymes, partially determine the mechanical properties and porosity of cell walls by demethylating pectin [74]. These enzymes have been shown to be involved in many developmental processes [75], and because they can strengthen or weaken the cell wall dependent on their mode of action and the environment, they are also likely important in stress response [75]. Consistent with a role for pectinesterases in cold tolerance, genes encoding pectinesterase and pectinesterase-like proteins were upregulated in E and downregulated in MH. 

Proteins involved in plant defense are also important for plant tolerance to biotic and abiotic stress. Genes encoding members of three protein families involved in plant immunity, SBT, GLR, and CRK, had increased expression in MH and decreased expression in E under cold stress (Table 2 and Table 3). SBTs are induced by pathogen infection, are secreted to the plant extracellular matrix, and function in pathogen recognition and immune priming [76,77]. SBTs may be involved in signal recognition in plant immunity. Plant GLRs play important roles in various plant-specific physiological processes, such as pollen tube growth, sexual reproduction, root meristem proliferation, internode cell elongation, stomata aperture regulation, and innate immunity and wound responses [78,79]. GLRs regulate ion fluxes across membranes, particularly the flux of calcium, a key second messenger in plant cell responses to stimuli [78]. GLRs of tomato mediate the cold acclimation-induced chilling tolerance by regulating apoplastic H_2_O_2_ production and redox homeostasis [80]. The increased expression of GLRs may aid ROS scavenging under cold stress. CRKs are a type of receptor-like kinase; they regulate various plant development processes and responses to biotic and abiotic stress [81]. Arabidopsis CRK28 was found to participate in root growth and epidermal cell differentiation and to fine-tune ABA signaling during germination and early root growth [81]. Mou et al. (2021) reported that *CaCRK5* was induced in pepper upon *Ralstonia solanacearum* infection and treatment with SA [82]. CRKs, as kinases, may affect plant hormone signaling and increase cold resistance. These proteins may be common to biotic and abiotic stress responses, and their functions may reflect common mechanisms underlying these responses. 

The common CSR DEGs are critical for elucidating the molecular mechanisms of *Anthurium* cold resistance, especially the DEGs upregulated in the cold-resistant cultivar E and downregulated in MH. These may explain the cold resistance of E and should be further studied to understand their function in *Anthurium* cold resistance. 

## 4. Materials and Methods

### 4.1. Plant Material and Stress Treatments

Two *Anthurium* cultivars, Elegang (E, cold-tolerant) and Menghuan (MH, cold-sensitive), were used in this study. The full-flowering plants were grown in a growth chamber for 3 days with a 12/12 h day/night cycle at 25/22 °C. For cold treatments, the plants were grown at 15 °C for 3 days, then the temperature was decreased to 6 °C for 3 days and then 4 °C for 3 days. The control plants were grown at 25/22 °C throughout the experiment. Samples were collected at 25/22 °C (control/CK), 3 days after the treatments at 6 °C and 4 °C. Three independent experimental replicates were performed, each consisting of three independent biological replicates. All the samples were immediately frozen in liquid nitrogen and stored at −80 °C for further use.

### 4.2. Determination of Enzyme Activity and MDA, Soluble Sugar, and Soluble Protein Contents

The collected samples were used for determining the physiological indexes in both cultivars. The activities of superoxide dismutase (SOD, EC 1.15.1.1) and peroxidase (POD, EC 1.11.1.7), and the contents of malondialdehyde (MDA), soluble sugar, and proline were measured according to Cai and Gao (2020) [83]. The content of soluble protein (cat no. BCAP-2-W) was measured using the kits provided by Suzhou Comin Biotechnology Co., Ltd. (Suzhou, Jiangsu, China).

### 4.3. RNA Extraction and cDNA Library Construction

Total RNA was isolated and purified using TRIzol reagent (Invitrogen, Carlsbad, CA, USA) following the manufacturer’s procedure. The amount and purity of RNA in each sample were quantified using a NanoDrop ND-1000 (NanoDrop, Wilmington, DE, USA). The RNA integrity was assessed by a Bioanalyzer 2100 (Agilent, Palo Alto, CA, USA) with RIN number >7.0 and confirmed by electrophoresis on a denaturing agarose gel. Poly (A) RNA was purified using Dynabeads Oligo (dT)25-61005 (Thermo Fisher, Waltham, MA, USA) and then fragmented into small pieces using the Magnesium RNA Fragmentation Module (NEB, Cat. e6150, Ipswich, MA, USA). The cDNA was then synthesized using the cleaved RNA fragments as template. After the addition of an ‘A’ tail, size selection of the cDNA was performed with AMPureXP beads. The cDNA library was constructed and subjected to 2 × 150 bp paired-end sequencing (PE150) on an Illumina Novaseq™ 6000.

### 4.4. De Novo Transcriptome Assembly and Analysis

Raw transcriptomic data generated by the Illumina Novaseq™ 6000 were preprocessed to remove the reads that contained adaptor contamination, low quality bases, and undetermined bases using the fastp [84] and perl scripts in-house. Then, the sequence quality was verified using fastp including the Q20, Q30, and GC-content of the clean data. All downstream analyses were based on clean data of high quality. De novo assembly of the transcriptome was performed with Trinity 2.4.0 [85]. Trinity groups transcripts into clusters based on shared sequence content, and a transcript cluster is very loosely referred to as a “gene”. The longest transcript in the cluster was chosen as the “gene” sequence (aka unigene). Salmon [86] was used to determine the expression level for unigenes by calculating transcripts per kilobase million (TPM) [87]. The differential expression analysis for two conditions or groups was performed using the R package edgeR [88]. The genes were considered differentially expressed between the treated and the control samples if |log_2_(foldchange)| > 2 and FDR (adjust *p*-value) < 0.001. All assembled unigenes were aligned against the non-redundant (Nr) protein database (http://www.ncbi.nlm.nih.gov/, accessed on 14 April 2021), SwissProt (http://www.expasy.ch/sprot/, accessed on 14 April 2021), Kyoto Encyclopedia of Genes and Genomes (KEGG) (http://www.kegg.jp/kegg/, accessed on 14 April 2021), and eggNOG (Evolutionary Genealogy of Genes: Non-supervised Orthologous Groups) (http://eggnogdb.embl.de/, accessed on 14 April 2021) databases using DIAMOND [89] with an E-value cut off of 10^−5^. Functional classification was performed using Gene Ontology (GO) (http://www.geneontology.org, accessed on 14 April 2021) by DIAMOND [89] with an E-value threshold of 10^−5^. The transcriptome sequence data were deposited in NCBI’s Gene Expression Omnibus repository (https://www.ncbi.nlm.nih.gov/geo, accessed on 21 May 2023), under accession number PRJNA973051.

### 4.5. Identification of Key Modules Related to Plant Cold Stress by WGCNA

WGCNA is a systems biology method for describing the correlation patterns among genes that have similar expression patterns in specific biological functions [47]. In this experiment, a total of 24,695 DEGs were used to construct weighted co-expression modules in the R package WGCNA (version 2.0), using the guidelines from the published tutorials (https://www.omicstudio.cn/analysis/wgcna?id=16, accessed on 14 October 2023). After sample clustering, the soft threshold of module analysis was determined using the scale independence and mean connectivity analysis of modules with different power values. The power value ranged from 1 to 30, then the values of scale independence and mean connectivity were calculated accordingly. The power value was calculated when the scale independence value was 0.85. To classify similar gene expression profiles into different gene modules, the average distance with a minimum size threshold of 30 and merge cut height of 0.25 were used to construct a hierarchical clustering dendrogram from the topological overlap matrix (TOM). Moreover, the associations of modules with conditions for the 18 samples were determined using the calculated module eigengenes and Pearson’s correlation coefficient values. The hub genes were selected by the cytohubba plug-in using the MCC algorithm and visualized by Cytoscape (version 3.7.1) software [90].

### 4.6. qRT-PCR Analysis

The RNA quality was checked by electrophoresis and the concentration was calculated according to the optical density (OD) at 260 nm and 280 nm using a Nanodrop 2000 (NanoDrop, Wilmington, DE, USA). The first-strand cDNA was reverse transcribed from 1 μg of the total RNA using the Evo M-MLVRT Kit (cat no. AG11711, Accurate Biology, Changsha, China). qRT-PCR was performed using the SYBR^®^ Green Premix ProTaq HS qPCR Kit (cat no. AG11718, Accurate Biology, Changsha, China) on the Roche LightCycle96 Real Time System (Roche, Switzerland). The total volume for each reaction was 10 μL, comprising 0.4 μL specific primers, 1 μL cDNA, 5 μL SYBR mixture, and 3.6 μL double-distilled H_2_O. The cycling program was as follows: 10 min at 95 °C, followed by denaturation at 95 °C for 15 s, annealing, and elongation at 60 °C for 30 s (40 cycles). Gene-specific primers were designed by Primer Premier 5.0 software (Table S11). The *actin* gene was used as an internal reference gene. The qRT-PCR expression analysis for each gene was performed using three technical replicates (with three biological replicates). The relative expression levels of genes were calculated using the 2^−ΔΔCT^ method [91]. The expression level of CK for E was used as a control; its value was set to 1. The relative expression levels of other samples (including the CK for MH) were determined relative to the control. In this way, we can compare the same gene across all samples of the two cultivars.

### 4.7. Statistical Analysis

The statistical analysis was performed using GraphPad Prism 8.0. The statistical significance was determined by a multiple *t*-test. Error bars represent SD (*n* = 3).

## 5. Conclusions

The main purpose of this study was to investigate the molecular mechanisms underlying the cold response in two *Anthurium* cultivars with contrasting cold tolerances, through analysis of morphological and physiological changes combined with analysis of the transcriptome data. Soluble protein content was identified as being vital for cold resistance. HSPs and HsfA, cell-wall-associated pectinesterases, and plant defense proteins are common components of cold-stress responses in both cultivars but have opposite expressions patterns. They may play important roles in *Anthurium* cold resistance and can represent targets for enhancing *Anthurium* cold tolerance. Finally, plant hormone signal transduction pathways, trehalose metabolism, and ribosomal proteins are important for the cold resistance of *Anthurium*. Our results suggest that proteins involved in protein synthesis (ribosomal proteins, HSPs, and HsfA), energy metabolism, and signal transduction pathways play essential roles in *Anthurium* cold resistance and should be further studied.

## Figures and Tables

**Table 1:** Assembly statistics for the *A. andraeanum* transcriptome.

Assembly Statistics	
Raw reads (average)	47,943,471.56
Total number of clean reads	840,806,398
Clean reads (average)	46,711,466.56
Clean bases (G)	117.67
Q20% (average)	98.48
Q30% (average)	95.09
GC% (average)	51.42
Number of transcripts (˃200 bp)	290,721
Number of unigenes	132,108
Minimum length (bp)	201
Maximum length (bp)	16,127
Average length (bp)	648
N50 length (bp)	1207
GC content (%)	44.84
Total assembled bases	85,547,816

**Table 2:** The common CSR-DEGs with opposite expression patterns in the cultivars E and MH under 6 °C stress.

Four Common CSR-DEGs Upregulated in E but Downregulated in MH under 6 °C Stress				
Gene_ID	Name	log_2_FC (E_6 vs. E_CK)	log_2_FC (MH_6 vs. MH_CK)	Annotation
TRINITY_DN43394_c2_g5	-	3.57	−4.21	-
TRINITY_DN56057_c0_g5	-	2.68	−3.36	uncharacterized protein LOC110667824 [*Hevea brasiliensis*]
TRINITY_DN42951_c1_g2	-	2.85	−2.61	-
TRINITY_DN52943_c0_g2	HSP17.4B	3.55	−2.42	17.4 kDa class III heat-shock protein [*Phoenix dactylifera*]
**Fifteen Common CSR-DEGs Upregulated in MH but Downregulated in E under 6 °C Stress**				
TRINITY_DN43741_c0_g2	-	−3.28	4.19	hypothetical protein L195_g017092, partial [*Trifolium pratense*]
TRINITY_DN35319_c2_g1	-	−2.04	3.91	uncharacterized protein LOC18094378 isoform X3 [*Populus trichocarpa*]
TRINITY_DN52379_c0_g2	BURP3	−3.76	9.79	BURP domain-containing protein 3 [*Phoenix dactylifera*]
TRINITY_DN39241_c0_g1	SBT1.8	−2.19	4.83	hypothetical protein B296_00025367 [*Ensete ventricosum*]
TRINITY_DN47085_c1_g1	SBT1.6	−2.05	4.74	hypothetical protein B296_00025367 [*Ensete ventricosum*]
TRINITY_DN42045_c2_g2	SBT1.9	−2.60	8.07	subtilisin-like protease SBT1.9 [*Phoenix dactylifera*]
TRINITY_DN35790_c0_g1	COL16	−5.34	3.72	uncharacterized protein LOC111300501 [*Durio zibethinus*]
TRINITY_DN37319_c0_g3	GLR2.1	−4.19	4.13	PREDICTED: glutamate receptor 2.7-like [*Elaeis guineensis*]
TRINITY_DN50258_c0_g4	-	−2.25	4.23	-
TRINITY_DN55351_c1_g2	CRK28	−4.24	4.48	hypothetical protein Ahy_B09g100047 isoform B [*Arachis hypogaea*]
TRINITY_DN32295_c0_g1	CRK6	−3.10	4.38	cysteine-rich receptor-like protein kinase 8 [*Phoenix dactylifera*]
TRINITY_DN54550_c0_g1	At3g47200	−2.38	3.13	PREDICTED: UPF0481 protein At3g47200-like [*Elaeis guineensis*]
TRINITY_DN56023_c0_g2	GLR2.2	−2.30	6.76	hypothetical protein AQUCO_03100069v1 [*Aquilegia coerulea*]
TRINITY_DN56023_c0_g1	GLR2.8	−2.03	6.54	Glutamate receptor 2.8 [*Vitis vinifera*]
TRINITY_DN49565_c0_g3	-	−3.55	3.72	-

**Table 3:** The common CSR DEGs with opposite expression patterns in cultivars E and MH under 4 °C stress.

Twenty-Seven Common CSR-DEGs Upregulated in E but Downregulated in MH under 4 °C Stress				
Gene_ID	Name	log_2_FC (E_4 vs. E_CK)	log_2_FC (MH_4 vs. MH_CK)	Annotation
TRINITY_DN45412_c0_g4	-	4.33	−4.71	PREDICTED: uncharacterized protein LOC105048415 [*Elaeis guineensis*]
TRINITY_DN56876_c2_g1	-	2.59	−3.07	-
TRINITY_DN43394_c2_g5	-	4.86	−3.71	-
TRINITY_DN52540_c1_g2	PME1	3.65	−4.19	pectinesterase 3 [*Jatropha curcas*]
TRINITY_DN56279_c2_g2	-	2.11	−3.64	-
TRINITY_DN46551_c2_g2	-	2.65	−3.66	-
TRINITY_DN35567_c1_g1	HSFA2B	3.09	−9.03	PREDICTED: heat shock factor protein HSF30-like [*Elaeis guineensis*]
TRINITY_DN37079_c1_g3	-	6.35	−3.61	putative nucleotidyltransferase, Ribonuclease H [*Rosa chinensis*]
TRINITY_DN36186_c0_g3	TIP4-3	2.79	−4.52	PREDICTED: probable aquaporin TIP4-3 [*Elaeis guineensis*]
TRINITY_DN36954_c1_g7	-	3.14	−2.65	-
TRINITY_DN56438_c1_g1	-	5.30	−2.24	-
TRINITY_DN51698_c0_g1	-	2.95	−6.30	-
TRINITY_DN51173_c0_g1	-	2.56	−3.04	-
TRINITY_DN33920_c1_g3	FAAH	2.87	−3.55	fatty acid amide hydrolase-like [*Ananas comosus*]
TRINITY_DN54886_c1_g3	-	2.74	−2.06	-
TRINITY_DN37706_c0_g1	PME1	3.18	−4.45	pectinesterase 3 [*Manihot esculenta*]
TRINITY_DN53485_c0_g2	-	4.42	−3.28	-
TRINITY_DN54221_c0_g4	-	2.09	−2.37	-
TRINITY_DN42775_c1_g1	MPE3	2.80	−3.45	pectinesterase 1-like [*Dendrobium catenatum*]
TRINITY_DN50024_c0_g1	-	2.61	−2.82	-
TRINITY_DN42193_c0_g1	-	2.66	−2.58	PREDICTED: uncharacterized protein LOC105803676 [*Gossypium raimondii*]
TRINITY_DN54864_c0_g4	-	2.61	−2.85	-
TRINITY_DN42951_c1_g2	-	2.13	−2.54	-
TRINITY_DN51579_c0_g3	-	2.18	−2.27	transcription factor MYBS3-like isoform X2 [*Quercus suber*]
TRINITY_DN45042_c0_g3	-	2.06	−3.21	hypothetical protein AQUCO_05400137v1 [*Aquilegia coerulea*]
TRINITY_DN52943_c0_g2	HSP17.4B	4.06	−2.12	17.4 kDa class III heat-shock protein [*Phoenix dactylifera*]
TRINITY_DN44614_c2_g1	ELIP1	5.51	−2.03	chloroplastic early light-induced protein [*Crocus sativus*]
**Eight Common CSR-DEGs Downregulated in E but Upregulated in MH under 4 °C Stress**				
TRINITY_DN46057_c0_g4	GPAT6	−4.12	7.57	Glycerol-3-phosphate 2-O-acyltransferase 6 [*Ananas comosus*]
TRINITY_DN55055_c2_g2	At1g61180	−4.25	3.37	probable disease resistance protein At5g63020 isoform X1 [*Citrus sinensis*]
TRINITY_DN44362_c1_g2	-	−2.86	3.86	-
TRINITY_DN49073_c0_g2	-	−2.71	2.24	-
TRINITY_DN55351_c1_g2	CRK28	−3.46	3.54	hypothetical protein Ahy_B09g100047 isoform B [*Arachis hypogaea*]
TRINITY_DN39795_c1_g3	-	−2.66	2.25	PREDICTED: uncharacterized protein LOC109022142, partial [*Juglans regia*]
TRINITY_DN45091_c1_g1	CYP704C1	−2.29	5.97	PREDICTED: cytochrome P450 704C1-like [*Elaeis guineensis*]
TRINITY_DN51899_c2_g3	-	−4.76	7.31	-

## Data Availability

The data supporting the conclusions of this manuscript have been displayed as figures and supplemental material, and will be made available by the authors, without undue reservation, to any qualified researcher.

## Supplementary Materials

The supporting information can be downloaded at https://www.mdpi.com/article/10.3390/ijms25010250/s1. Figure S1: GO categories of 132,018 unigenes. Figure S2: KEGG category of 132,108 unigenes. Figure S3: Number of all DEGs. (A) DEGs from comparison of cultivars. (B) DEGs from comparison of cold treatment vs. control. Figure S4: KEGG analysis of cultivar-only DEGs in common 9132 DEGs. Figure S5: GO analysis of common DEGS for the same treatment between two cultivars. Figure S6: Scatter plots for KEGG pathway enrichment analysis of DEGs in the four key modules. (A) KEGG analysis of DEGs in the turquoise module; (B) KEGG analysis of DEGs in the greenyellow module; (C) KEGG analysis of DEGs in the tan module; (D) KEGG analysis of DEGs in the yellow module. Only the top 20 KEGG pathways are shown in the plots. Figure S7: Relative gene expression analysis of 16 randomly selected DEGs by qRT-PCR. Comparison of qRT-PCR with fold-change values calculated using transcriptome data from E and MH cultivars. The relative expression level is presented on the left *y*-axis, and the fold-change values from the transcriptome data are shown on the right *y*-axis. The relative expression levels of 16 common DEGs were investigated using qRT-PCR. The results from three replicates were analyzed using the 2^−ΔΔCt^ method and normalized to the *actin* housekeeping gene. The data shown are the means of three biological replicates (*n* = 3), and the bar represents the standard deviation (SD) for each mean value. Table S1: Overview of the RNA sequencing reads for the 18 *A. andraeanum* leaf samples. Table S2: All enriched GO terms for the 132,018 unigenes. Table S3: DEGs between cold-tolerant and cold-susceptible cultivars under the same temperature conditions. Table S4: A list of cold-responsive genes in E and MH. Table S5: All information about the 9132 common DEGs. Table S6: The enriched GO terms for the four key modules. Table S7: The top 20 hub genes of the four key modules. Table S8: The log2(fold-change) of DEGs involved in plant hormone signal transduction pathways. Table S9: The 25 trehalose-related DEGs. Table S10: The 78 DEGs encoding ribosomal proteins. Table S11: Primer sequences for qRT-PCR analysis of DEGs.
