# Supplementary material for: Comparative Physiological and Transcriptome Analyses of Tolerant and Susceptible Cultivars Reveal the Molecular Mechanism of Cold Tolerance in Anthurium andraeanum
Source: Int J Mol Sci. 2023 Dec 23;25(1):250. doi: 10.3390/ijms25010250 (PMC10779044; doi:10.3390/ijms25010250)
Supplement: Supplementary file 1 [file ijms-25-00250-s001.zip › Wu et al. Figure S1-7.pdf]

Figure S1

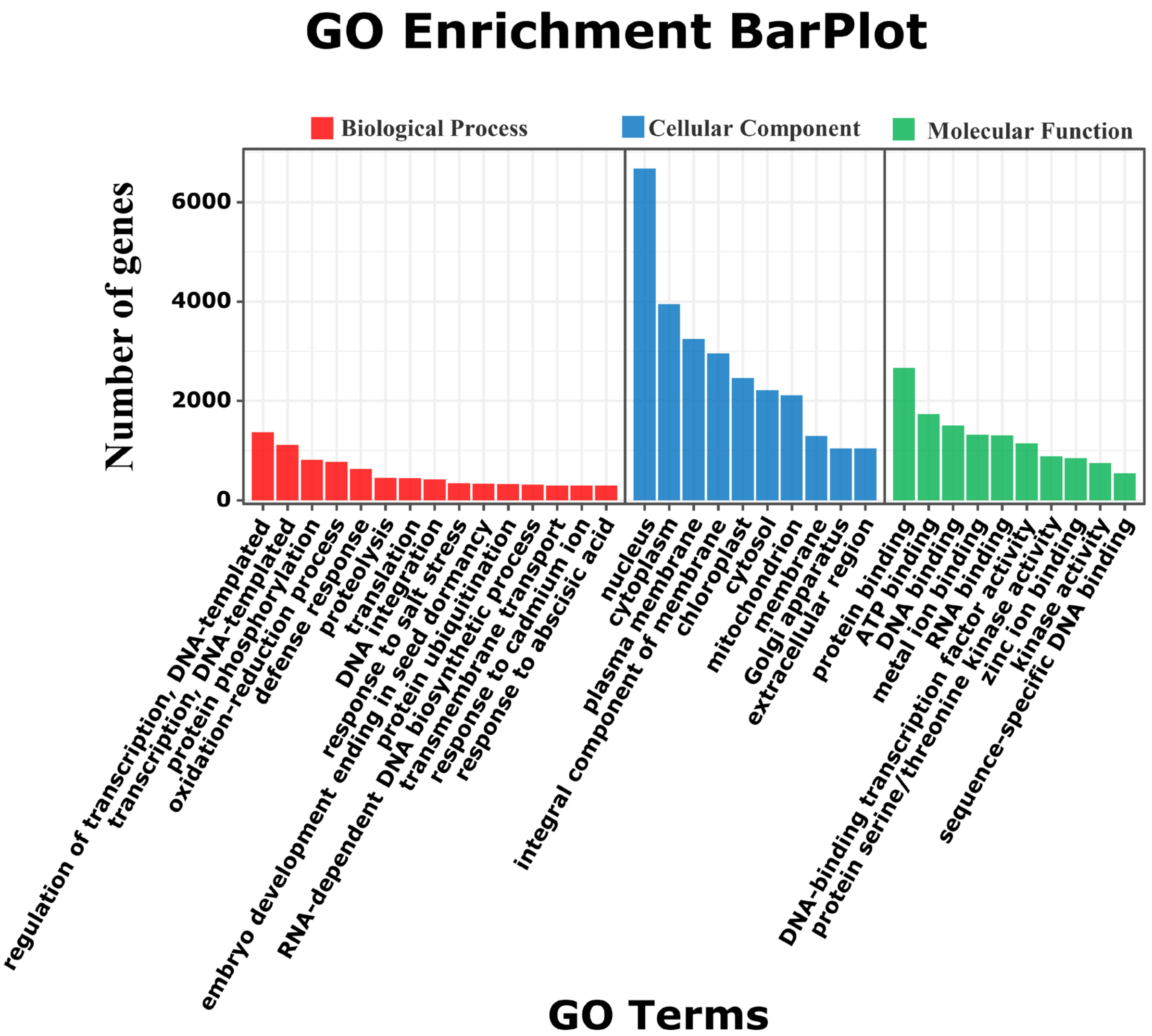
GO Enrichment BarPlot
Biological Process
Cellular Component
Molecular Function
Number of genes
6000
4000
2000
0
regulation of transcription, DNA-templated
transcription, DNA-templated
protein phosphorylation
oxidation-reduction process
defense response
proteolysis
translation
DNA integration
response to salt stress
embryo development ending in seed dormancy
protein ubiquitination
RNA-dependent DNA biosynthetic process
transmembrane transport
response to cadmium ion
response to abscisic acid
nucleus
cytoplasm
plasma membrane
integral component of membrane
chloroplast
cytosol
mitochondrion
membrane
Golgi apparatus
extracellular region
protein binding
ATP binding
DNA binding
metal ion binding
RNA binding
DNA-binding transcription factor activity
protein serine/threonine kinase activity
zinc ion binding
kinase activity
sequence-specific DNA binding
GO Terms

Figure S2

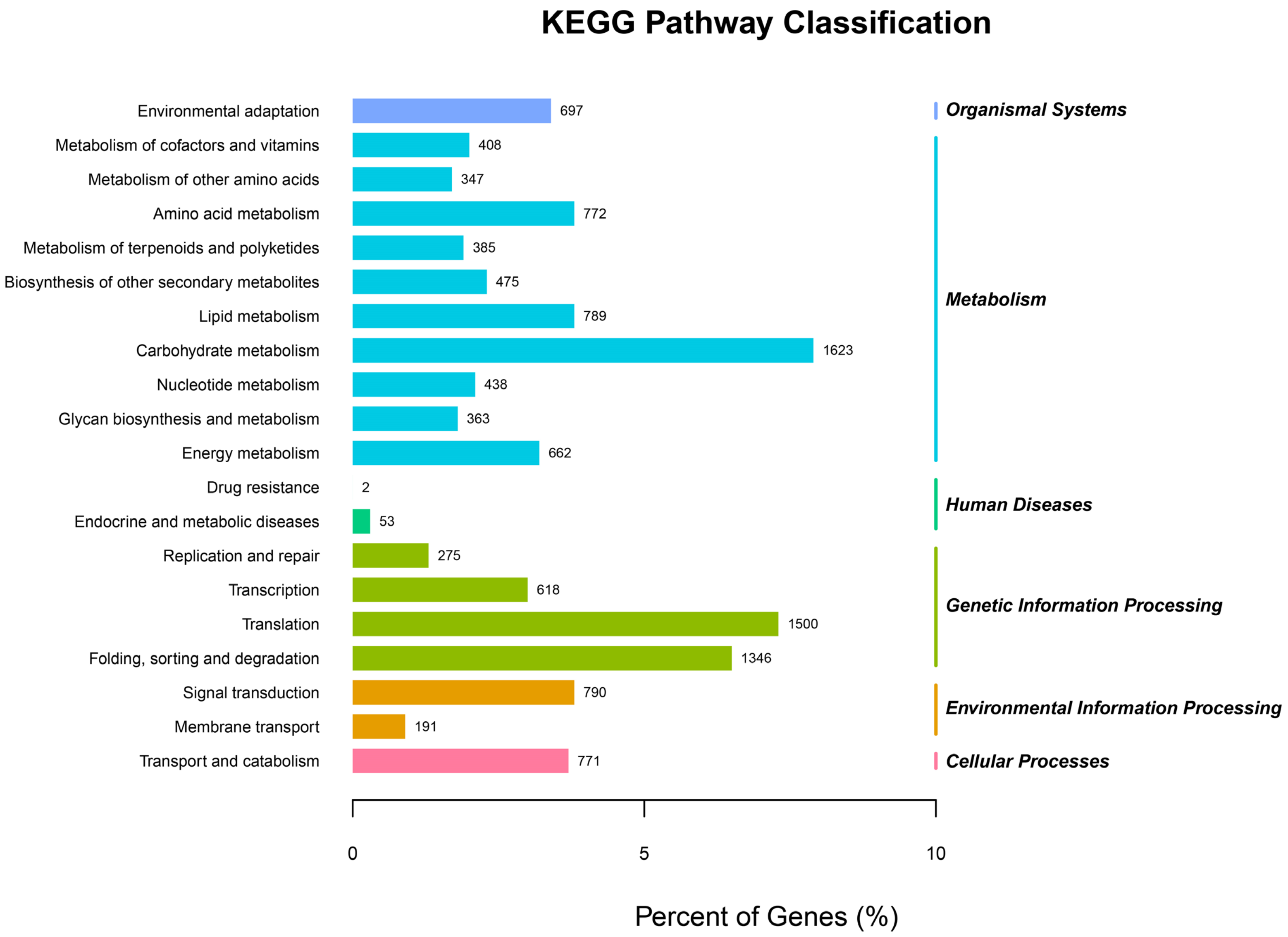

KEGG Pathway Classification
Environmental adaptation
Metabolism of cofactors and vitamins
Metabolism of other amino acids
Amino acid metabolism
Metabolism of terpenoids and polyketides
Biosynthesis of other secondary metabolites
Lipid metabolism
Carbohydrate metabolism
Nucleotide metabolism
Glycan biosynthesis and metabolism
Energy metabolism
Drug resistance
Endocrine and metabolic diseases
Replication and repair
Transcription
Translation
Folding, sorting and degradation
Signal transduction
Membrane transport
Transport and catabolism
697
408
347
772
385
475
789
1623
438
363
662
2
53
275
618
1500
1346
790
191
771
Organismal Systems
Metabolism
Human Diseases
Genetic Information Processing
Environmental Information Processing
Cellular Processes
0
5
10
Percent of Genes (%)

Figure S3

(A)

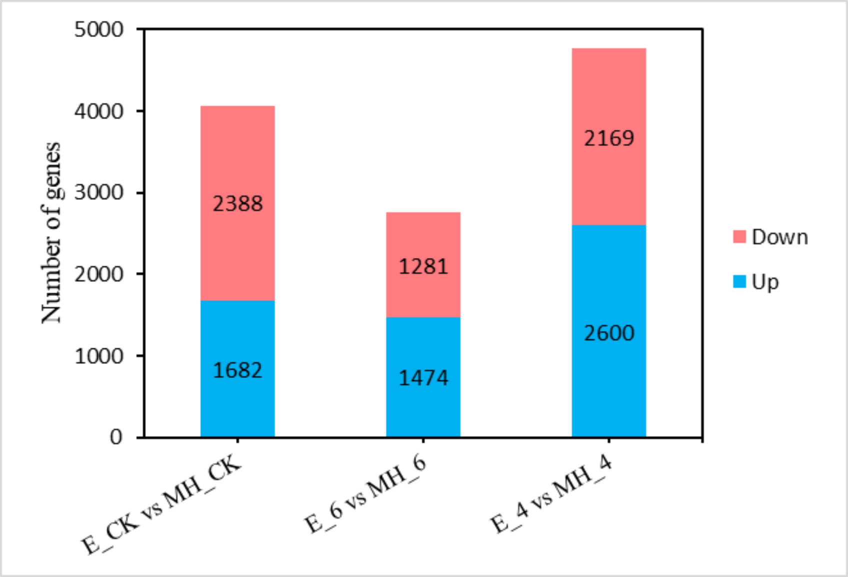

5000
4000
3000
2000
1000
0
Number of genes
2388
1682
1281
1474
2169
2600
Down
Up
E_CK vs MH_CK
E_6 vs MH_6
E_4 vs MH_4

(B)

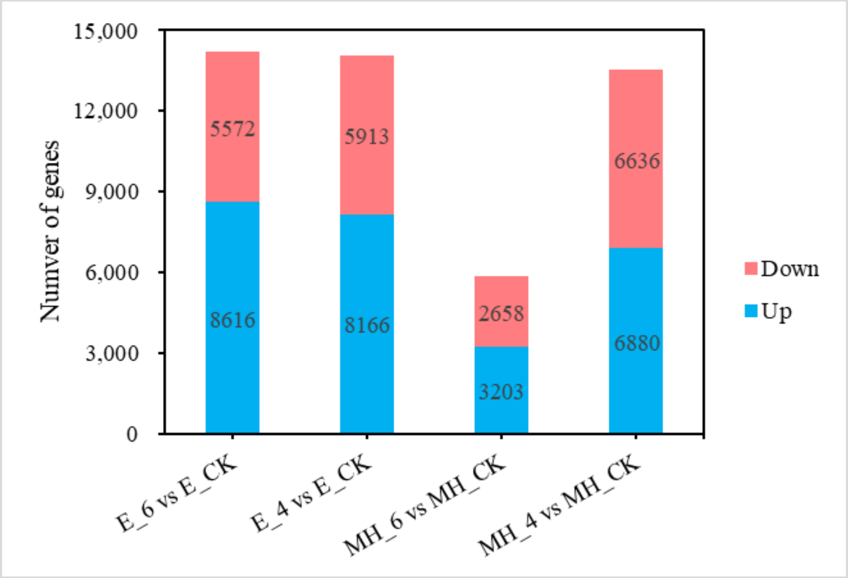

15,000
12,000
9,000
6,000
3,000
0
Numver of genes
5572
8616
5913
8166
2658
3203
6636
6880
Down
Up
E_6 vs E_CK
E_4 vs E_CK
MH_6 vs MH_CK
MH_4 vs MH_CK

Figure S4

E_6 vs E_CK only:

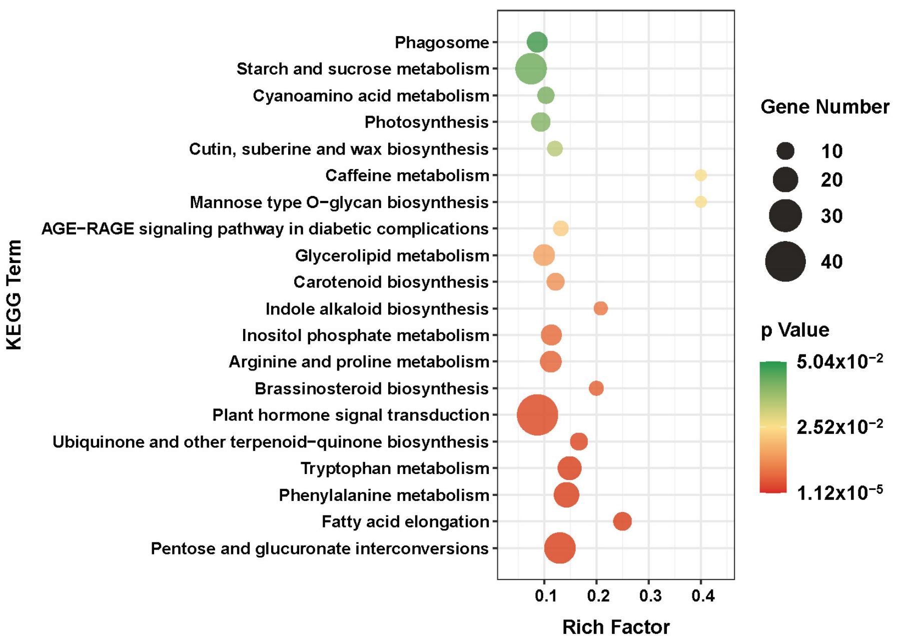

MH_6 vs MH_CK only:

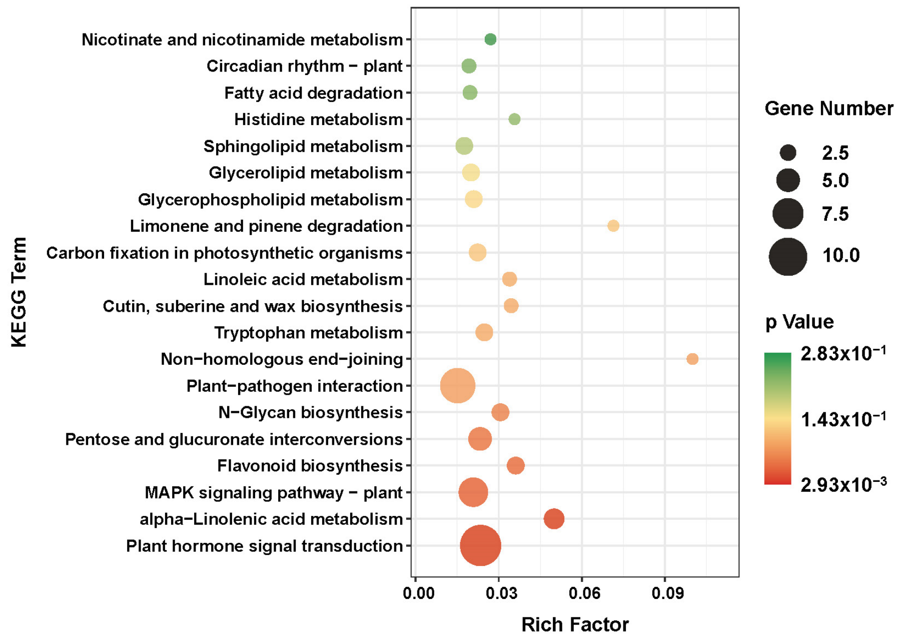

E_4 vs E_CK only:

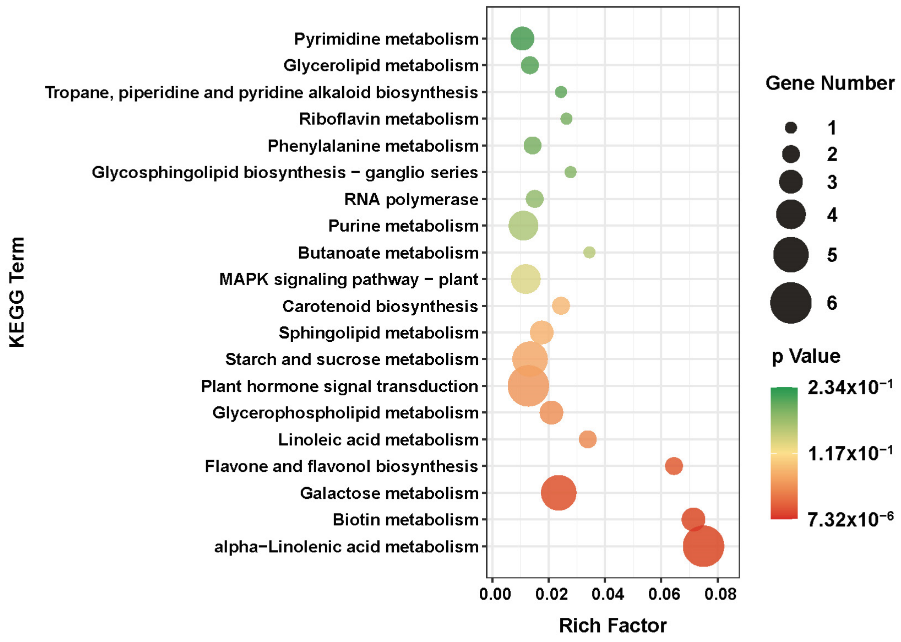

MH_4 vs MH_CK only:

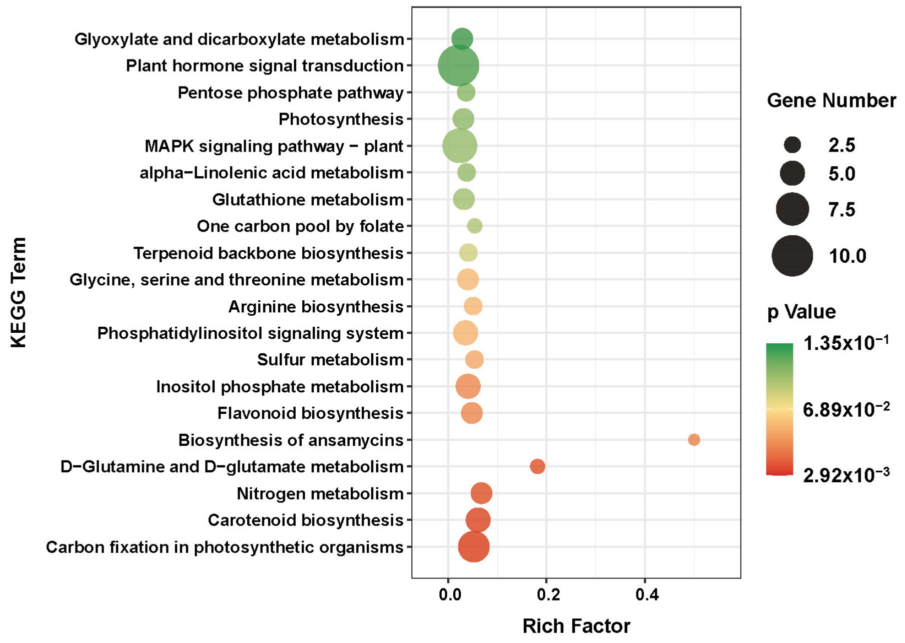

Figure S5

Up: E_6 vs E_CK & MH_6 vs MH_CK

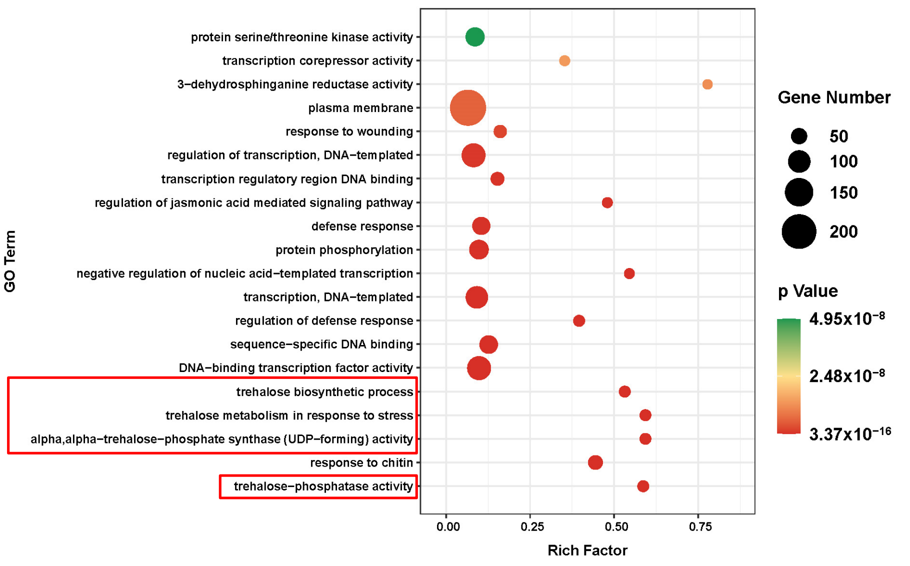

Down: E_6 vs E_CK & MH_6 vs MH_CK

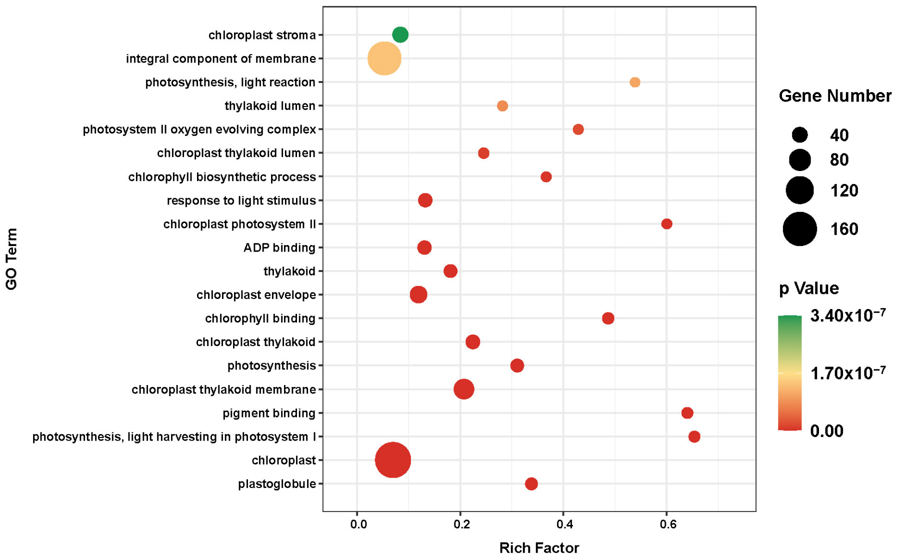

Up: E_4 vs E_CK & MH_4 vs MH_CK

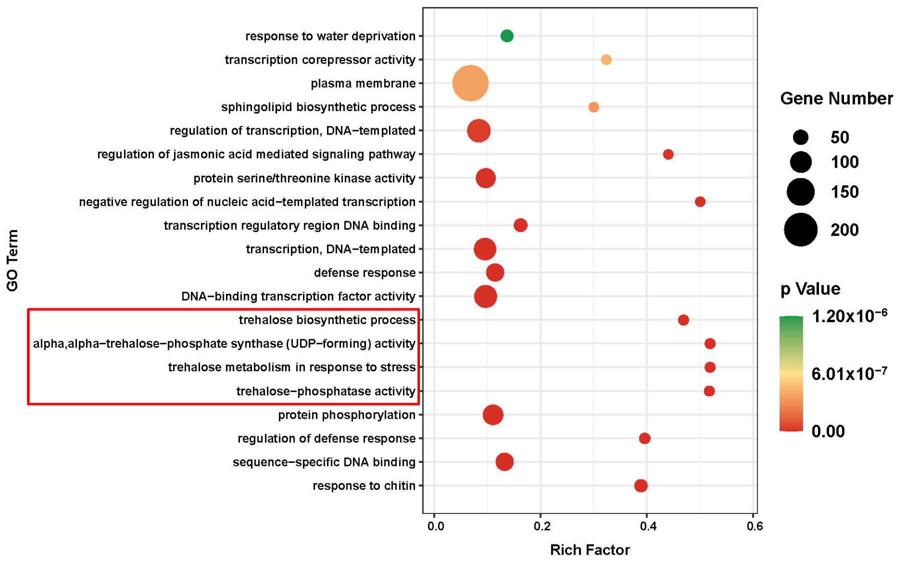

Down: E_4 vs E_CK & MH_4 vs MH_CK

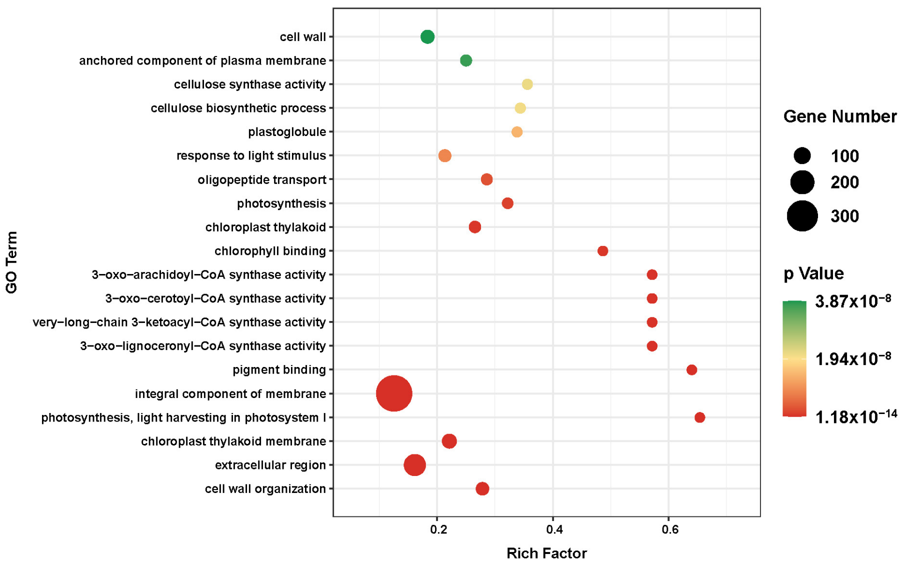

Figure S6

(A) Module turquoise:

(B) Module greenyellow:

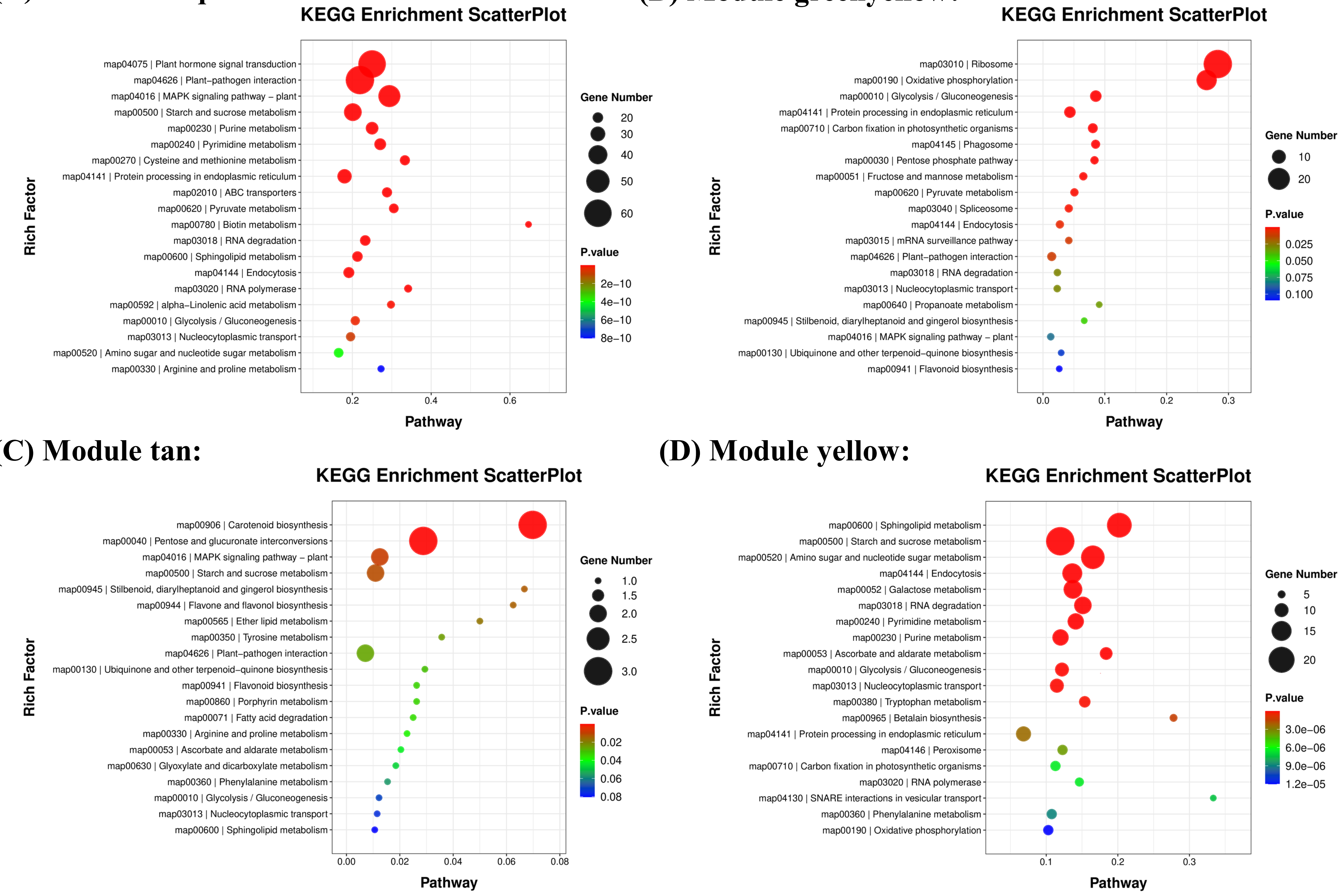

Figure S7

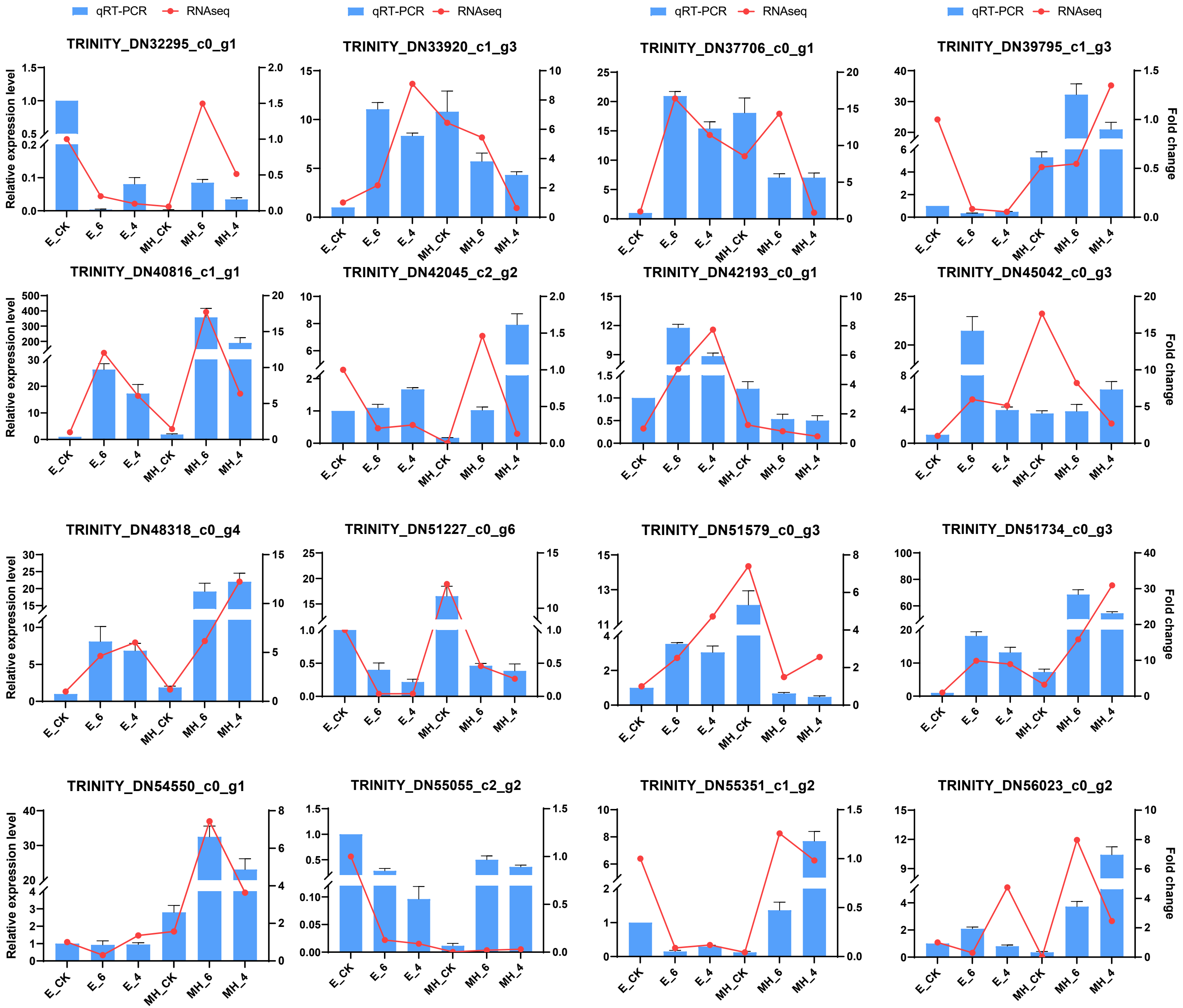

qRT-PCR
RNAseq
TRINITY_DN32295_c0_g1
TRINITY_DN33920_c1_g3
TRINITY_DN37706_c0_g1
TRINITY_DN39795_c1_g3
TRINITY_DN40816_c1_g1
TRINITY_DN42045_c2_g2
TRINITY_DN42193_c0_g1
TRINITY_DN45042_c0_g3
TRINITY_DN48318_c0_g4
TRINITY_DN51227_c0_g6
TRINITY_DN51579_c0_g3
TRINITY_DN51734_c0_g3
TRINITY_DN54550_c0_g1
TRINITY_DN55055_c2_g2
TRINITY_DN55351_c1_g2
TRINITY_DN56023_c0_g2
Relative expression level
Fold change
E_CK
E_6
E_4
MH_CK
MH_6
MH_4
